# Supplementary material for: Multi-omics integration accurately predicts cellular state in unexplored conditions for Escherichia coli
Source: Nat Commun. 2016 Oct 7;7:13090. doi: 10.1038/ncomms13090 (PMC5059772; doi:10.1038/ncomms13090)
Supplement: Supplementary Information — Supplementary Figures 1-28, Supplementary Tables 1-3, Supplementary Methods, Supplementary References. [file ncomms13090-s1.pdf]

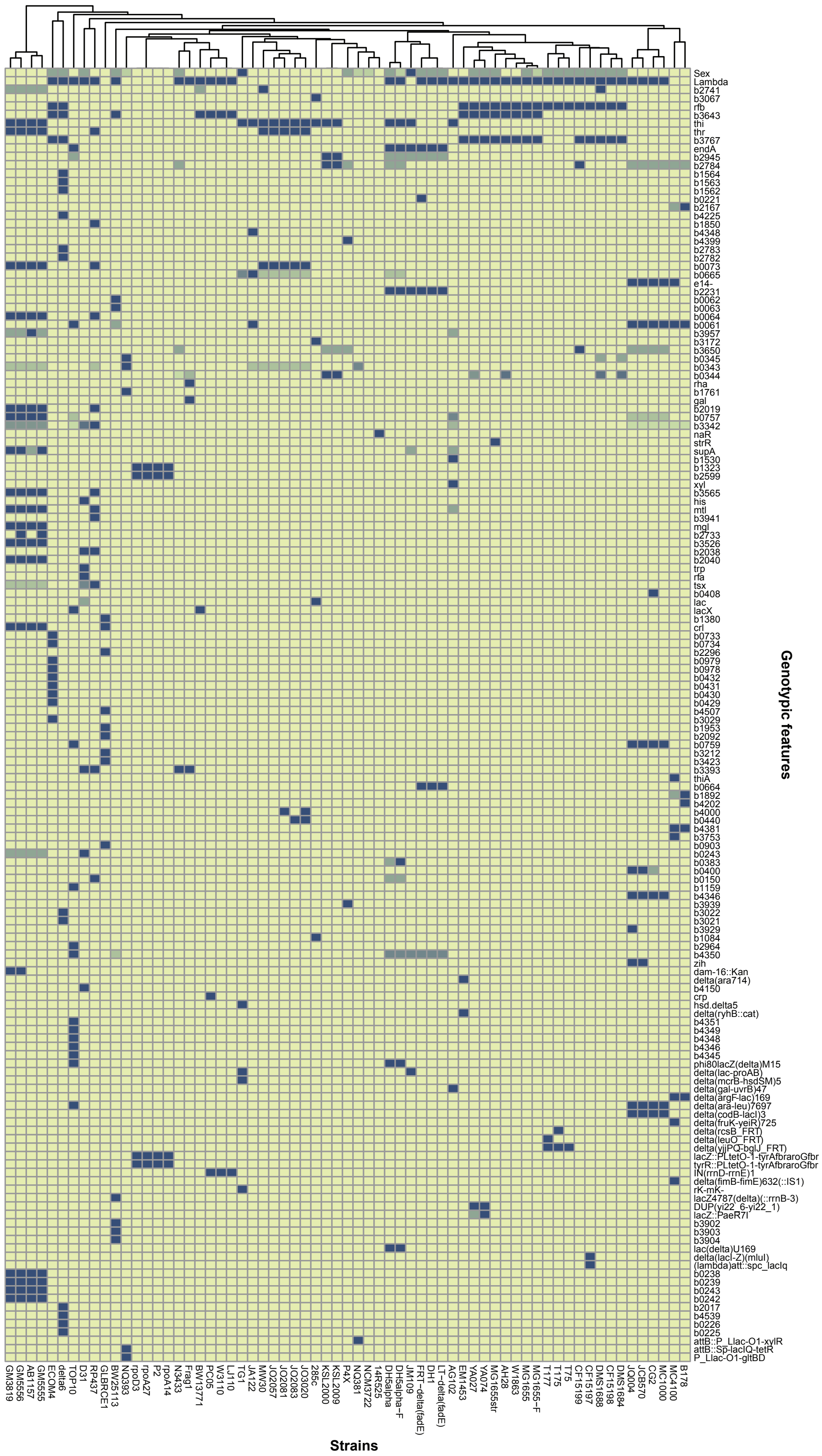

Supplementary Figure 1: Genotype-based strain representation. We characterized the 65 strains and 112 media with 154 and 120 features, respectively. Blue, gene deletion; grey, gene mutation; yellow, wild-type genotype.

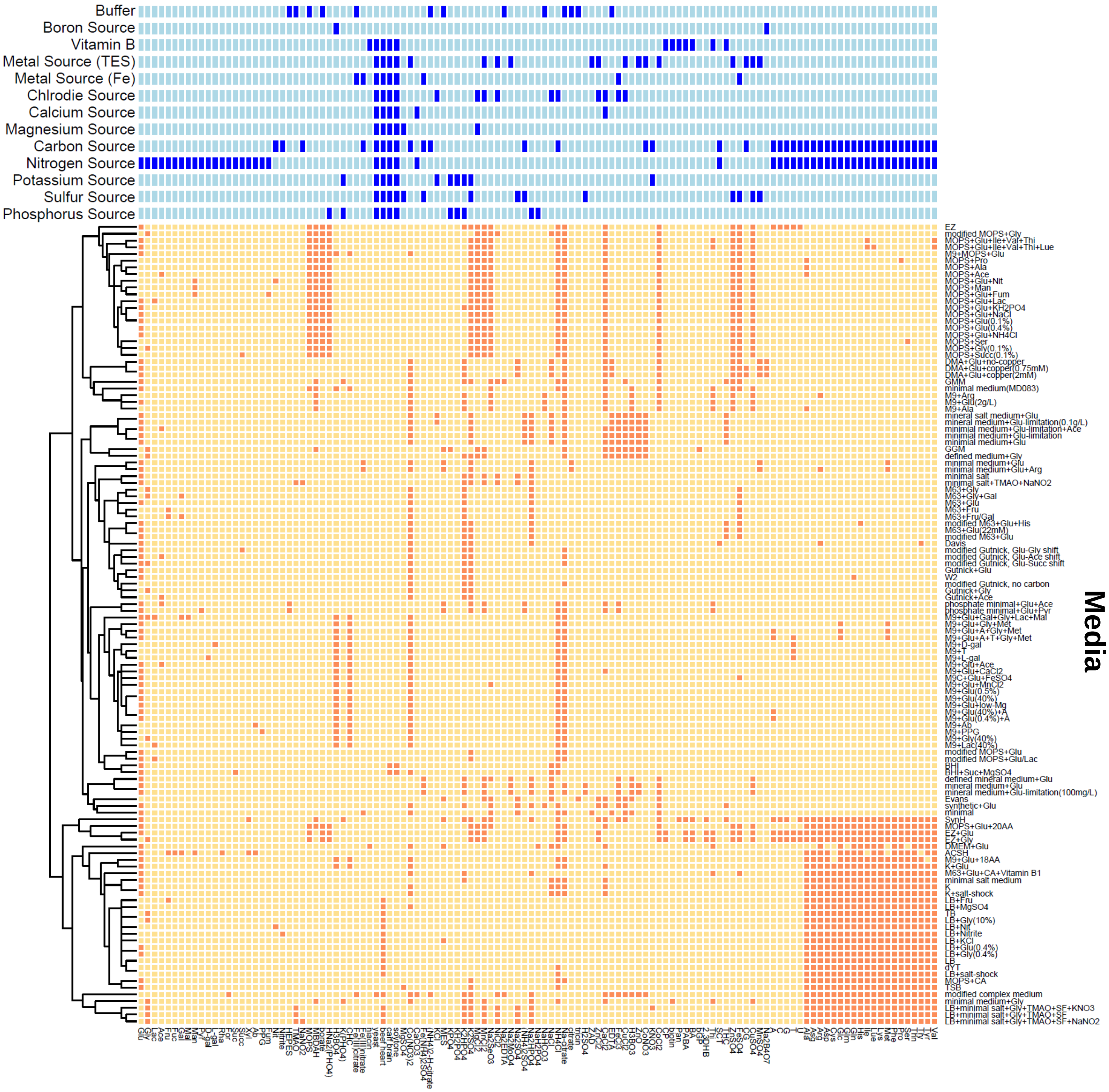

Supplementary Figure 2: Medium representation. We characterized the 65 strains and 112 media with 154 and 120 features, respectively. The blue table (top) groups together the chemical features by their major chemical sources.

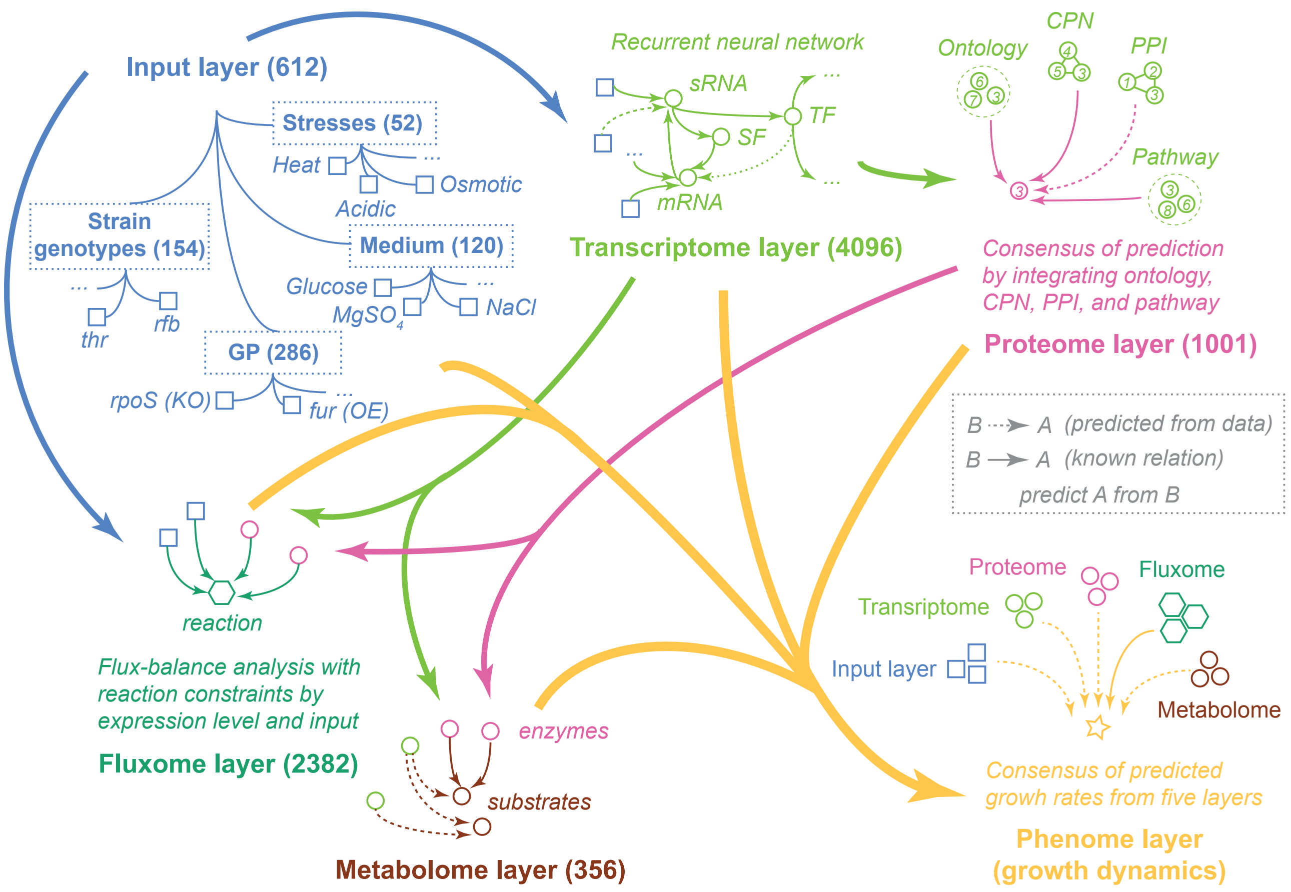

Supplementary Figure 3: The genome-scale model is composed of multiple layers with interdependencies. The input layer (612 features) is grouped into four categories: strain genotype (154), medium composition (120), stress (52), and genetic perturbation (GP, 286). To predict expression and growth dynamics in a novel condition, a recurrent neural network first predicts the genome-scale transcriptional expression (4096 transcripts) that it is then used together with information from gene ontology, co-expressed protein network (CPN), protein-protein interaction network (PPI), and other pathways to predict protein expression (1001 proteins). Concentrations of 356 metabolites are predicted from the transcriptome and proteome layers. Fluxes of 2382 reactions are predicted using Flux Balance Analysis (FBA) with constraints from the input, transcriptome and proteome layer. The growth rate is then inferred by consensus of predictions made from all five layers (including the input layer). KO; knock-out, OE; over-expression, SF; sigma factor, TF; transcription factor

A

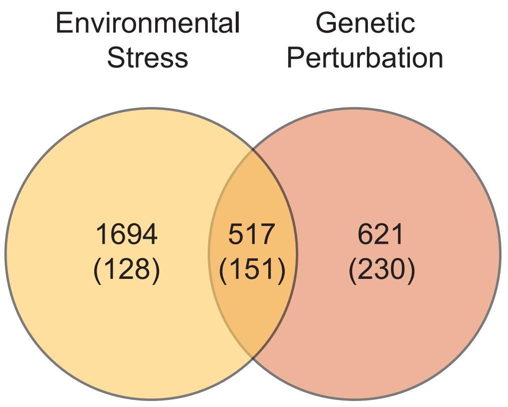

B

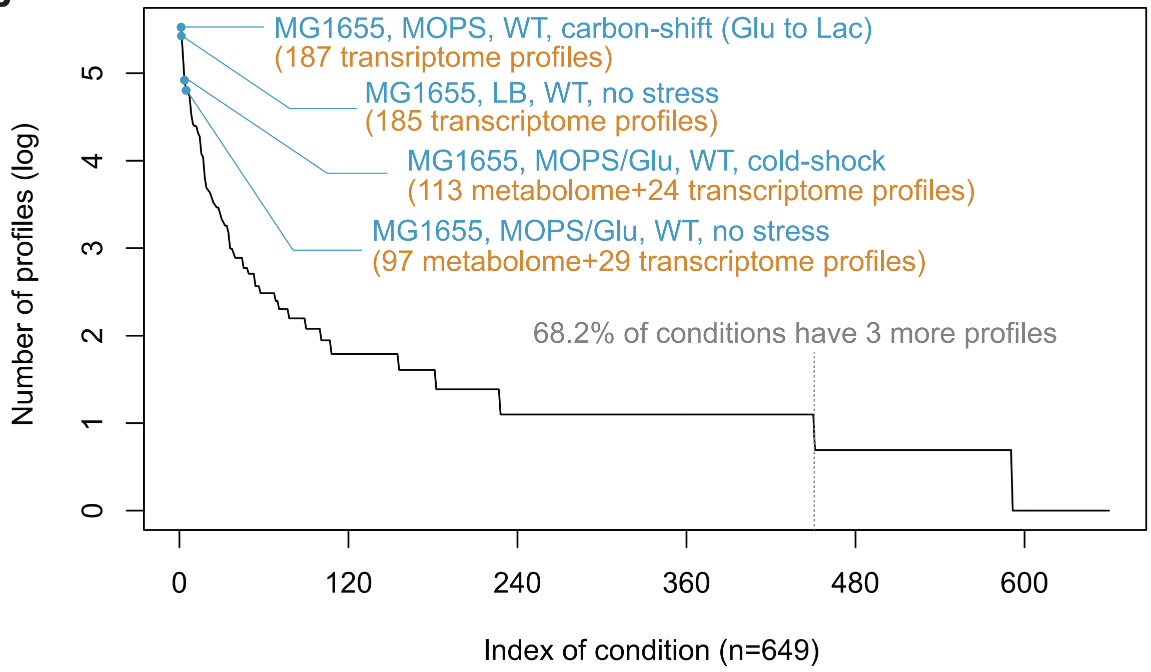

Supplementary Figure 4: Profiles per layer. (A) the Venn diagram of size of profile set for environmental perturbation studies and genetic perturbation studies (transcriptome layer). (B) the size of profile set for each condition ordered by its size.

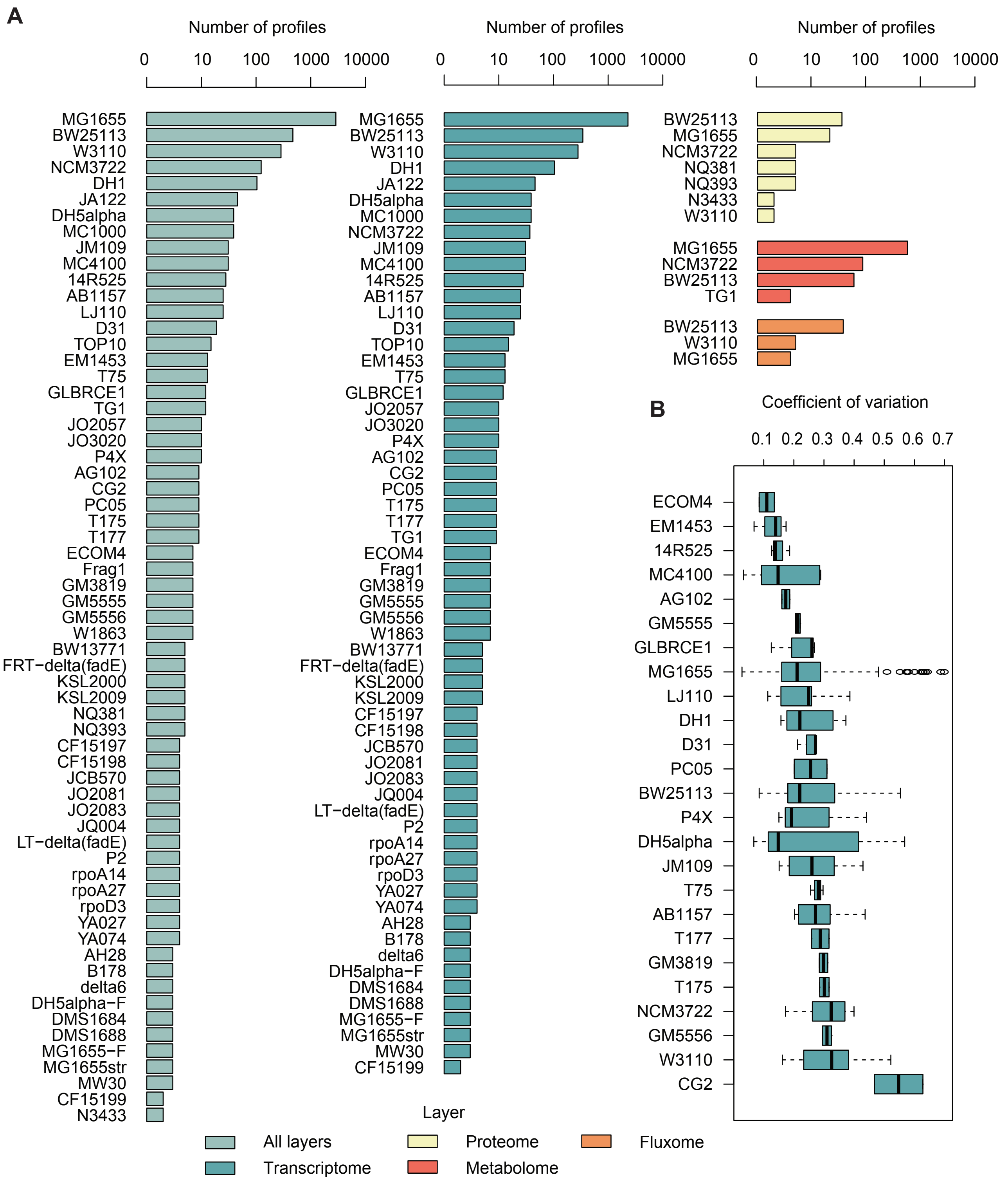

Supplementary Figure 5: (A) Profile distribution for all strains in Ecomics. (B) Variance of expression profiles for strains used in Ecomics.

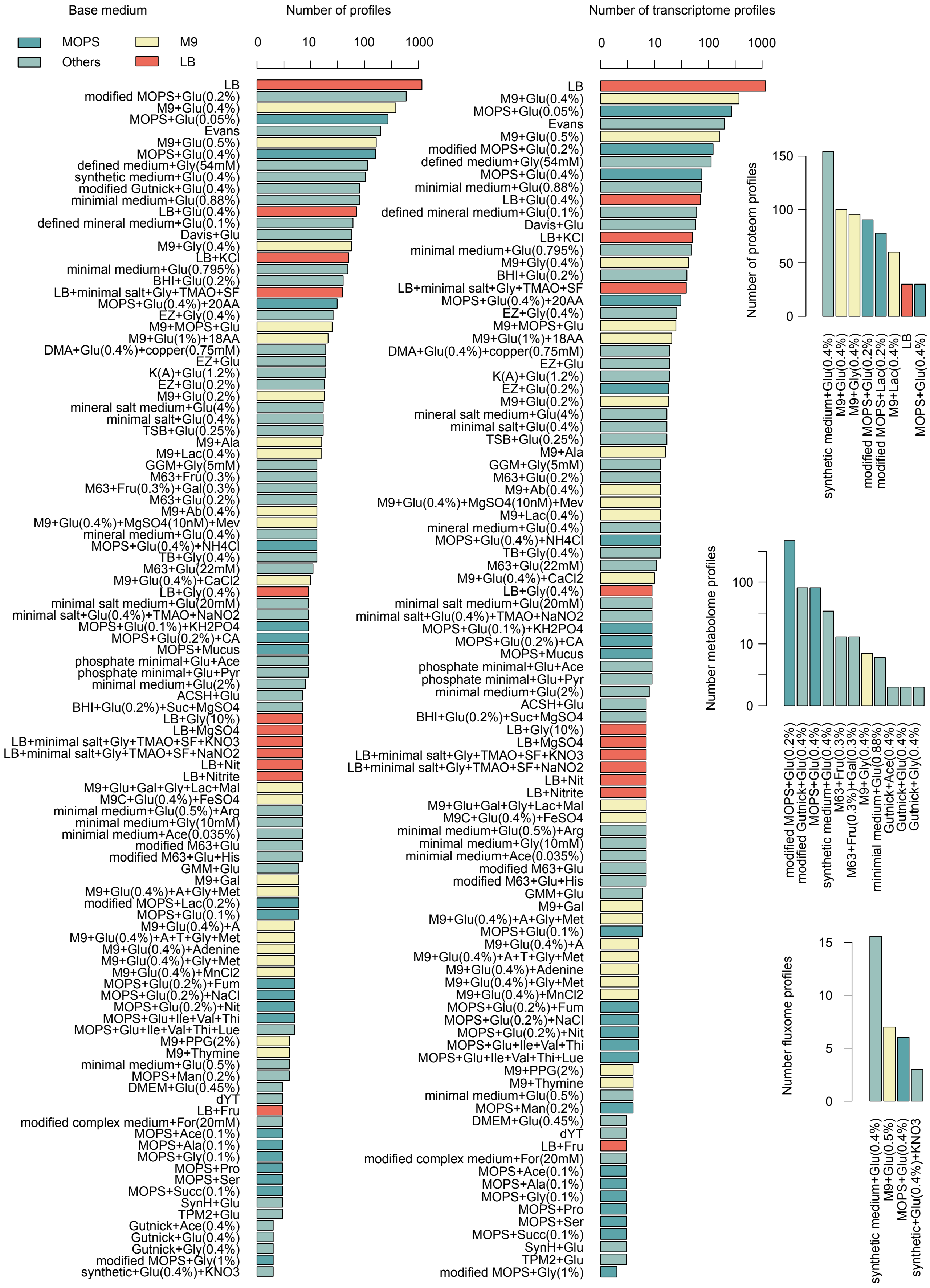

Supplementary Figure 6: Profile distribution for all media in Ecomics.

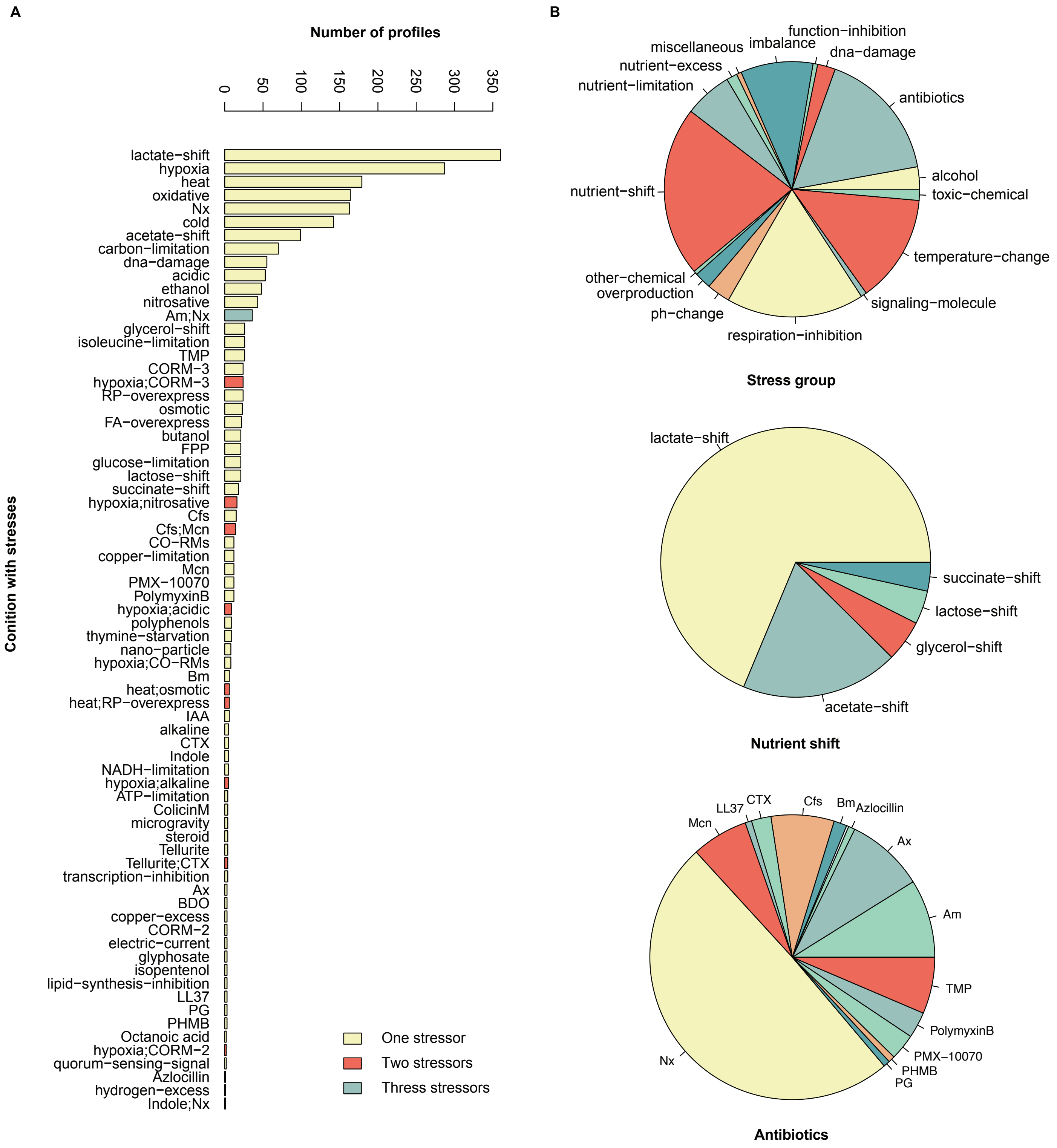

Supplementary Figure 7: Profile distribution for stresses present in Ecomics. (A) 75 conditions (65 single stressors) ordered by the number of their profiles. (B) 65 stresses are grouped into 16 categories (top pie chart), two groups of nutrient shift (middle pie chart) and antibiotics (bottom pie chart). Nx, norfloxacin; Am, ampicillin; Ab, arabinose; RP, recombinant protein; FA, free fatty acid; Cfs, cefsulodin; Mcn, mecillinam; Bm, biocyclomycin; PG, penicillin-G.

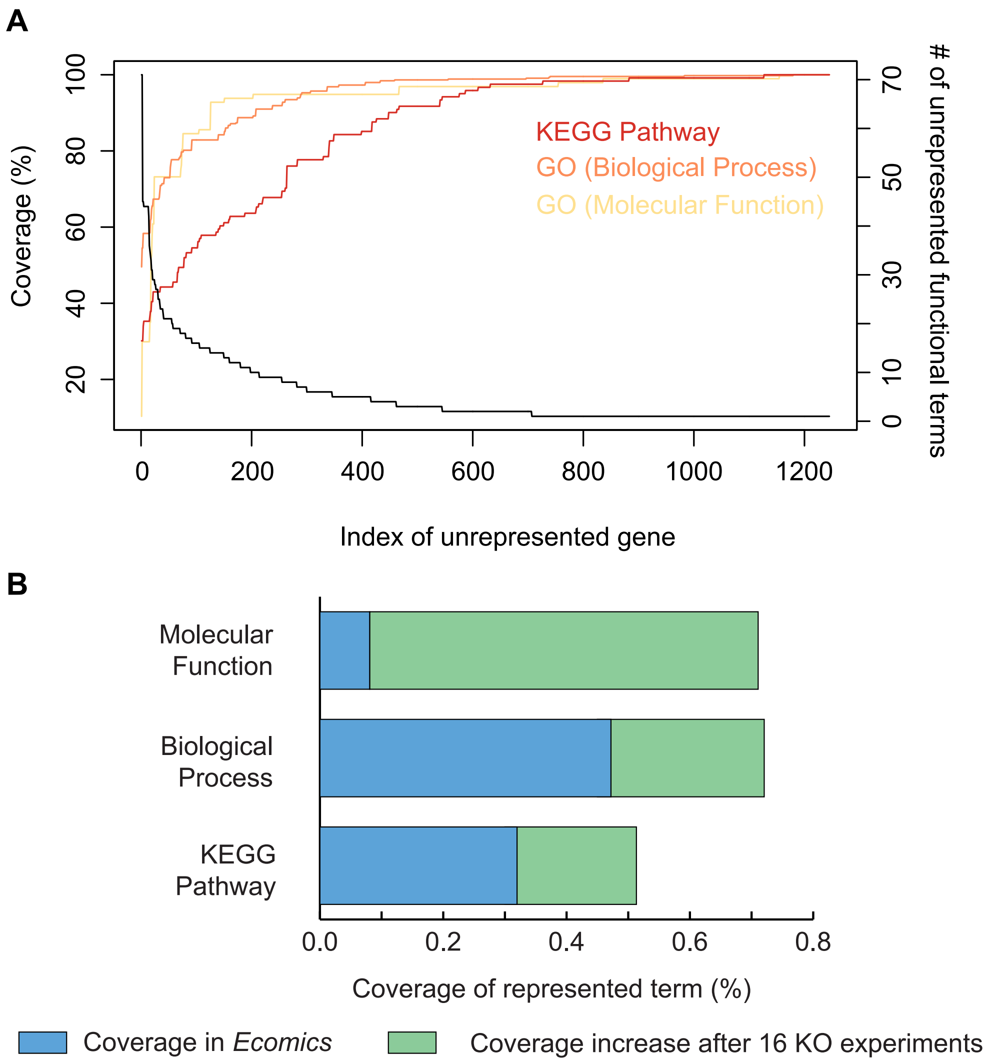

Supplementary Figure 8: Targeted experimentation for enriched GO and KEGG coverage. (A) Increase in GO and KEGG coverage by adding genes having most unrepresented terms in the compendium. (B) The coverage increase from 16 genes selected in an adaptive way. A gene that has most unrepresented terms is selected. Then the set of represented terms are updated and next gene is selected based on it. This procedure repeats until 16 genes are selected.

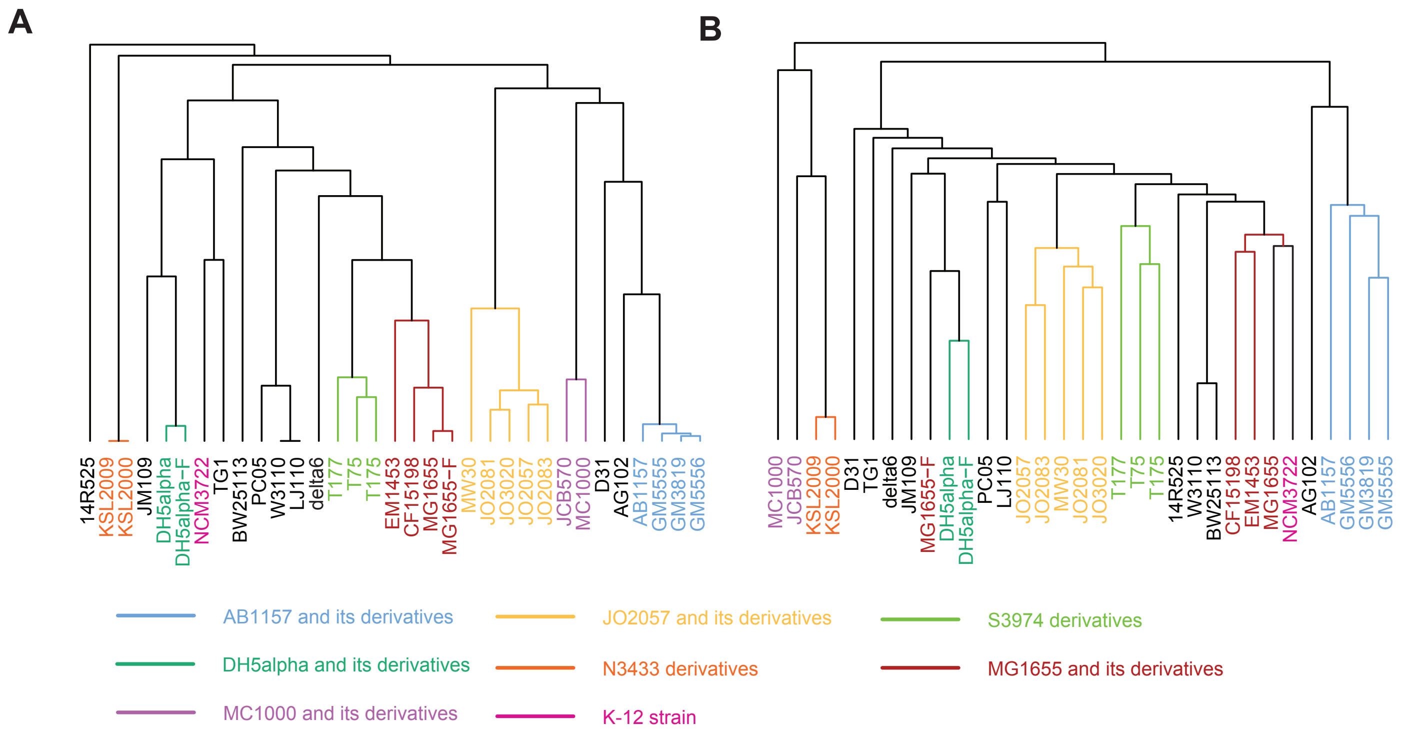

Supplementary Figure 9: Strain ontology in Ecomics. (A) Strain ontology based on genetic features. (B) Merged ontology based on both genotypic features and expression profiles (equal weight).

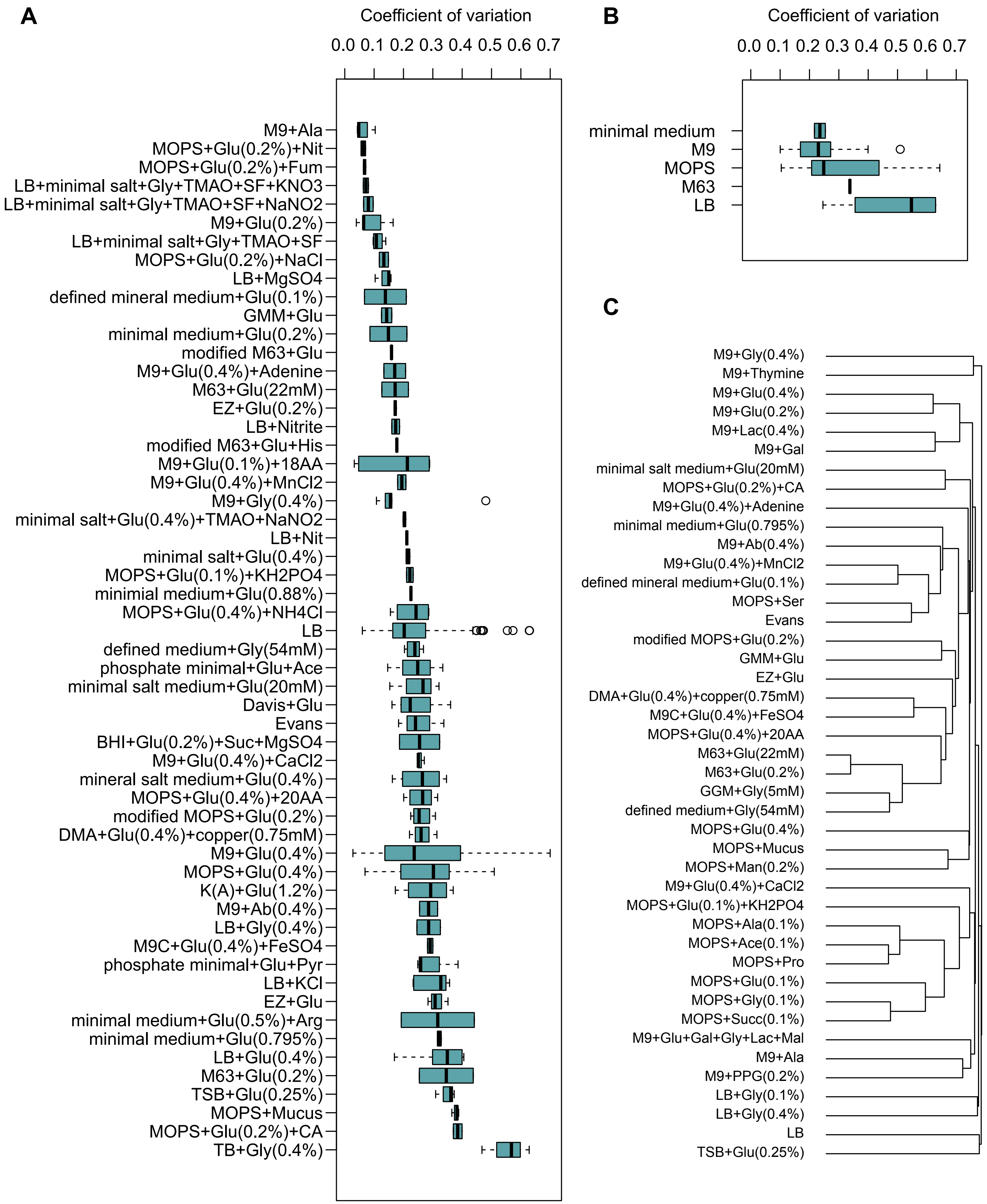

Supplementary Figure 10: (A) Media expression variability using all profiles. (B) Media expression variability using MG1655 strain without stress or genetic perturbation. (C) Media ontology based on gene expression profiles.

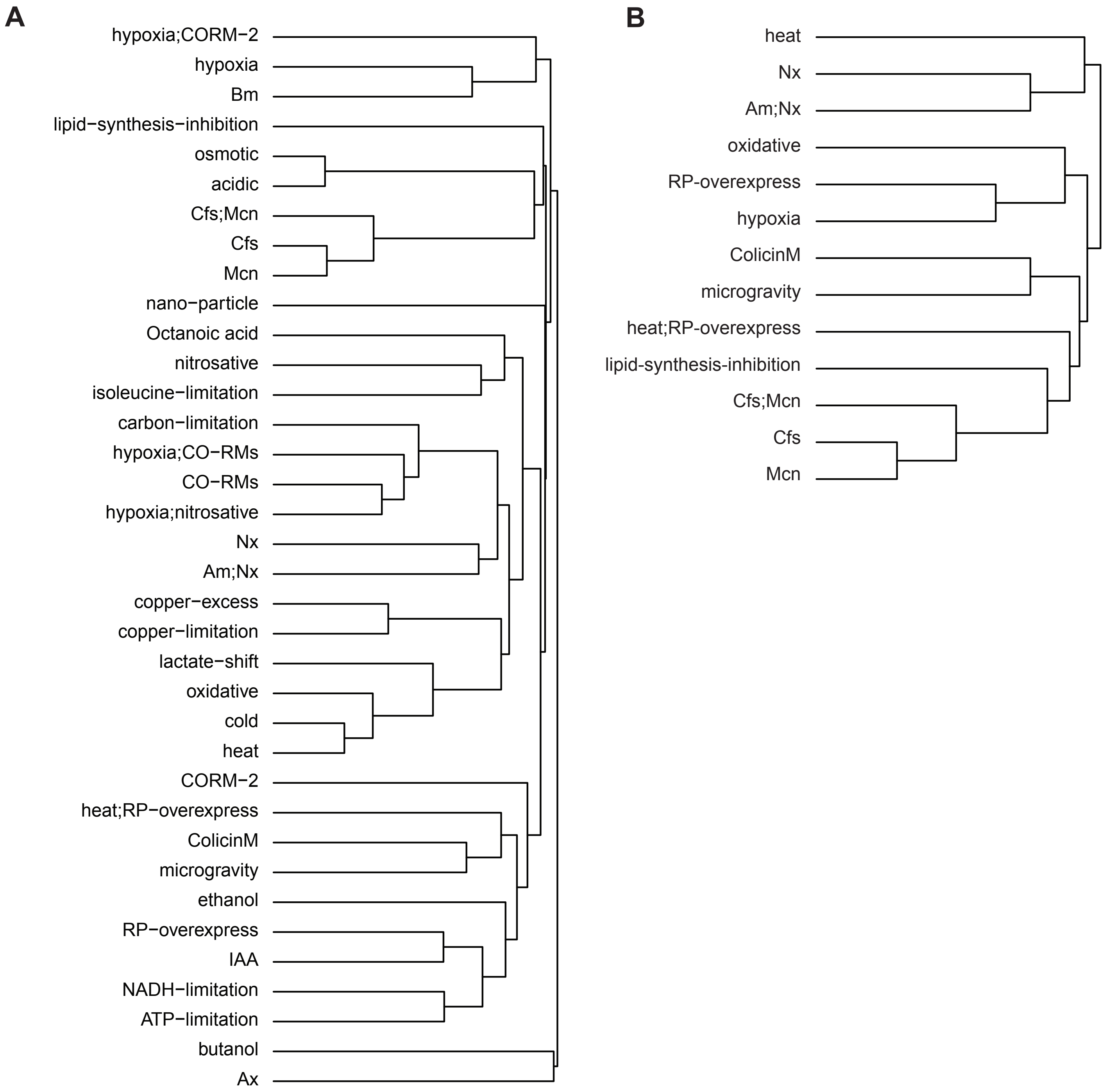

Supplementary Figure 11: Stress ontology in Ecomics. (A) Stress ontology using all profiles. (B) Stress ontology for MG1655 with LB medium. Bm, biocyclomycin; Cfs, cefsulodin; Mcn, mecillinam; Nx, norfloxacin; Am, ampicillin; RP, recombinant protein; IAA, indole-3-acetic acid; Ax, amoxicillin, CO-RM, carbon monoxide-releasing molecule

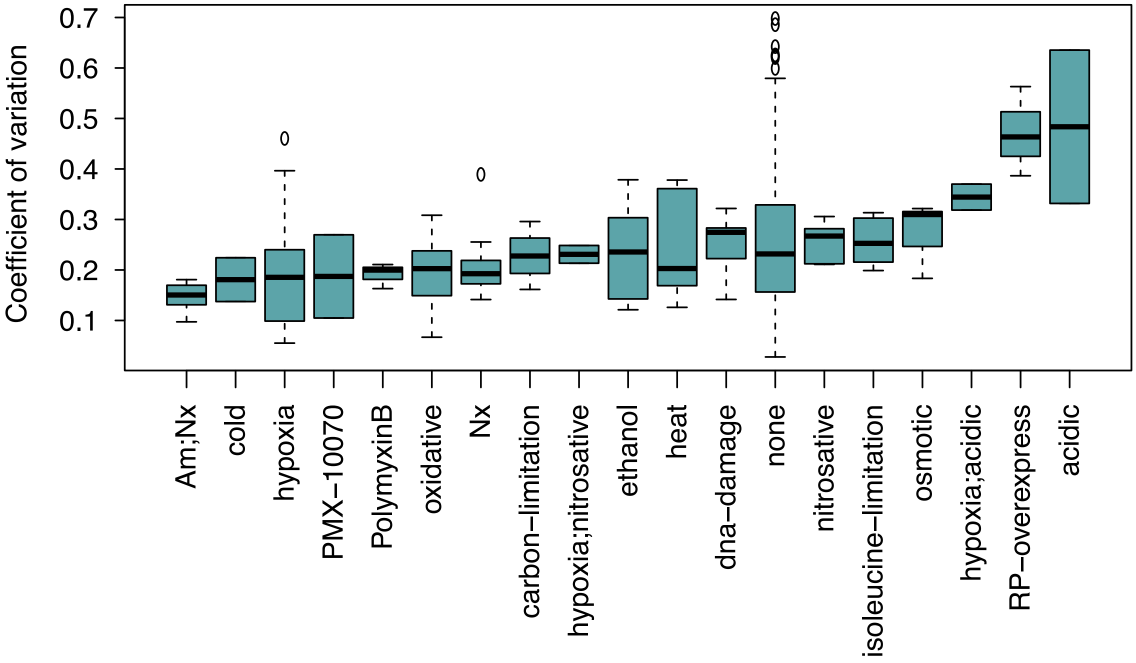

Supplementary Figure 12: Variance of expression profiles for stresses used in Ecomics.

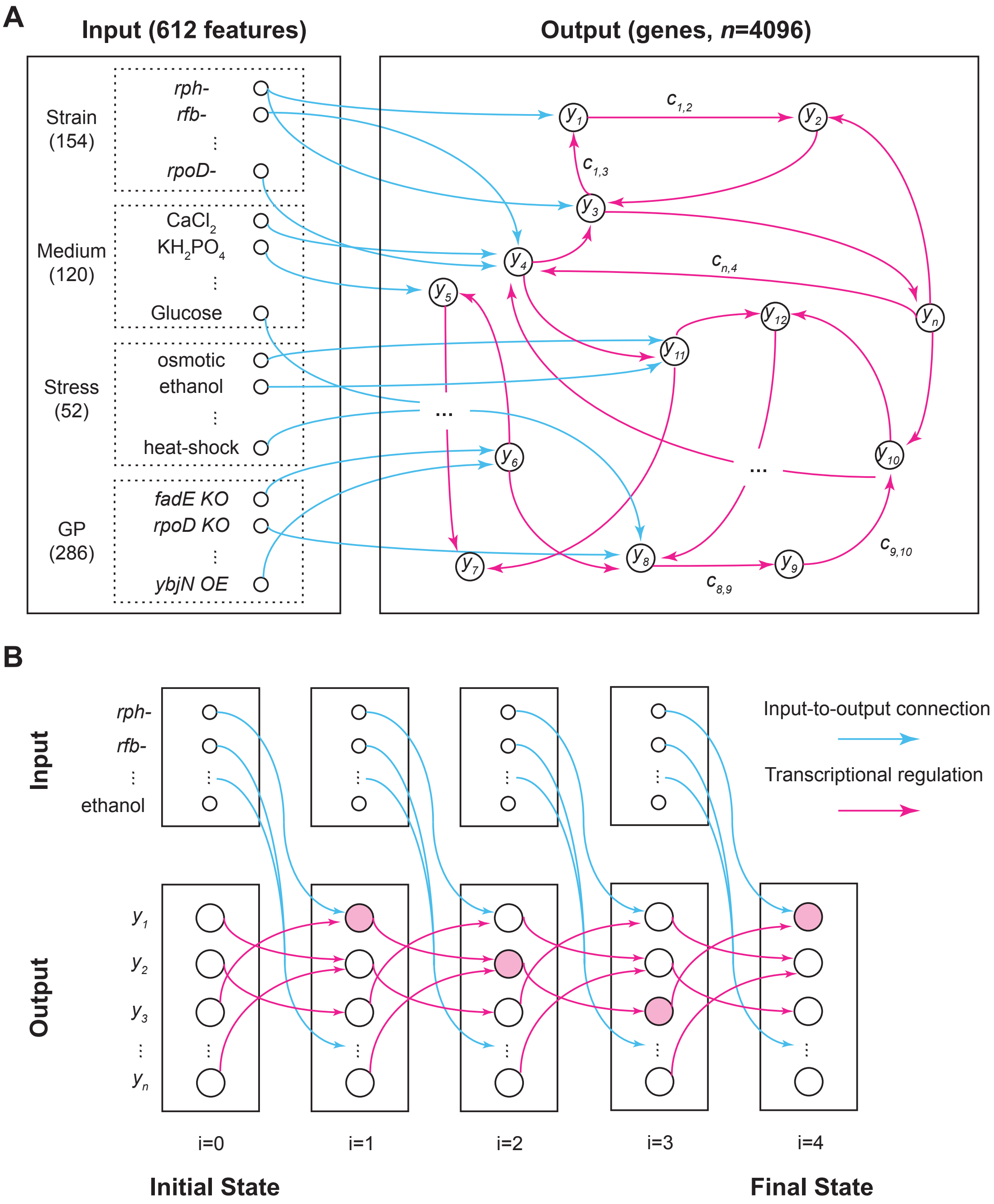

Supplementary Figure 13: Transcriptome prediction through a Recurrent Neural Network architecture. (A) A Recurrent neural network links environmental and genetic conditions to gene expression and growth rate. The network uses sigmoid activation functions and Lasso regression on the Ecomics compendium. (B) An unfolded RNN, where memory depth is 4. The node in red shows how the state of g_1 at time step 1 propagates and finally results in feed-back loop in its original node at time step 4.

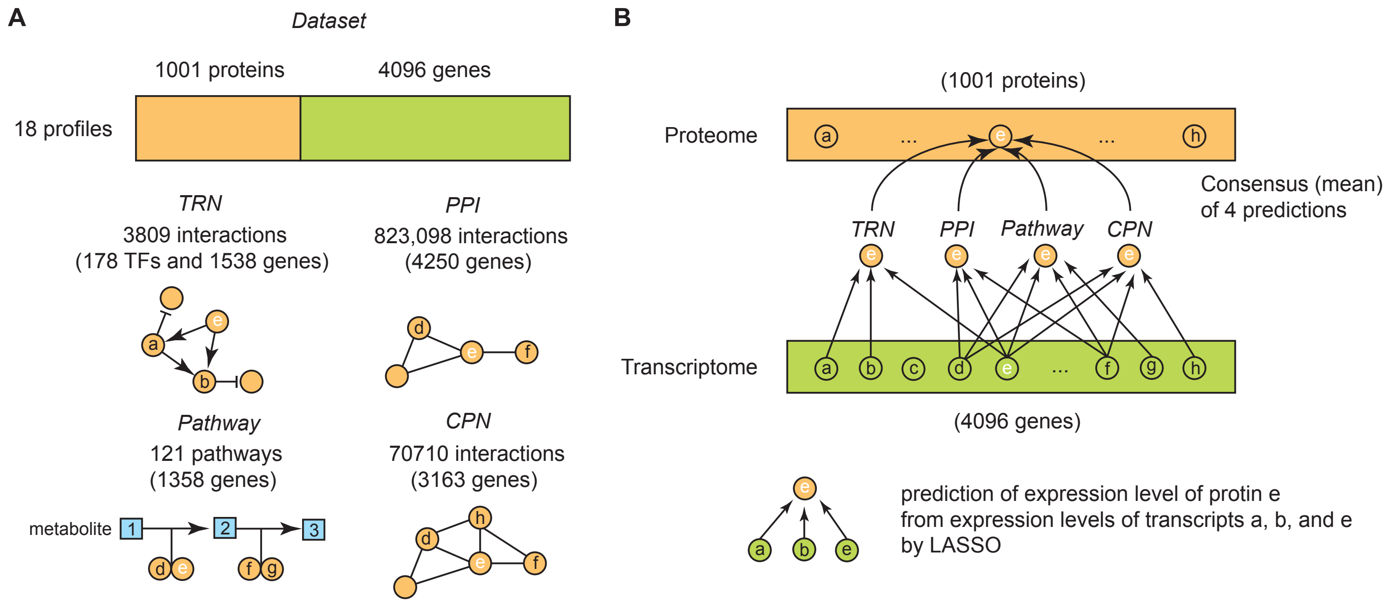

Supplementary Figure 14: (A) The dataset and four sources to predict proteome layer. (B) Prediction of protein expression levels from transcriptional expression levels. Protein expression level of e is predicted from consensus (mean) of four predictions where each is constructed from either of four network sources. For example, e is related with a and b and thus, its protein expression is predicted from transcriptional expression levels of a, b, and e by LASSO.

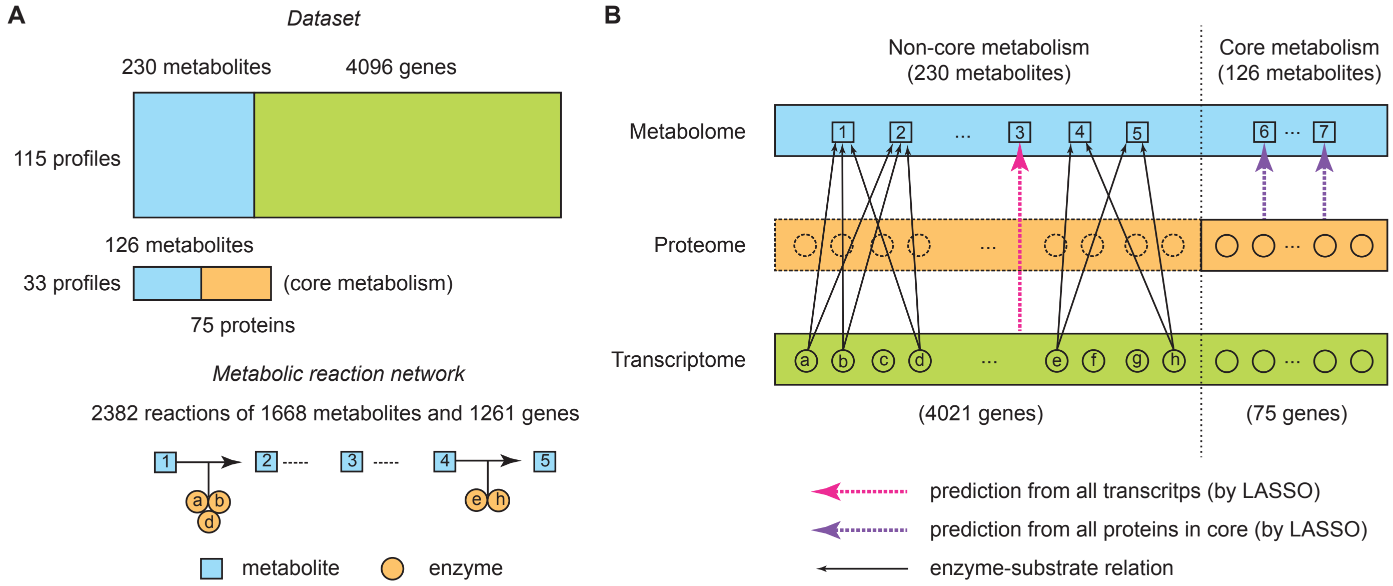

Supplementary Figure 15: (A) The dataset and metabolic reaction network (B) Prediction of metabolite concentrations from proteome and transcriptome layer. Metabolic concentration is predicted from two layers of transcriptome and proteome. Metabolites in core (metabolite 6 and 7) are predicted from all enzymes (using their protein expression levels) in core metabolism. For non-core metabolites, they are predicted from transcriptome and for metabolites having enzyme-substrate relations, they are predicted from transcriptional expression levels of related enzymes. For example, metabolites of 1 and 2 are catalyzed by enzymes of a, b, and d, and thus each concentration is predicted from mRNA expression levels of a, b, and d. For metabolites having no such relation information (e.g. metabolite 3), each of them is predicted from all genes by LASSO.

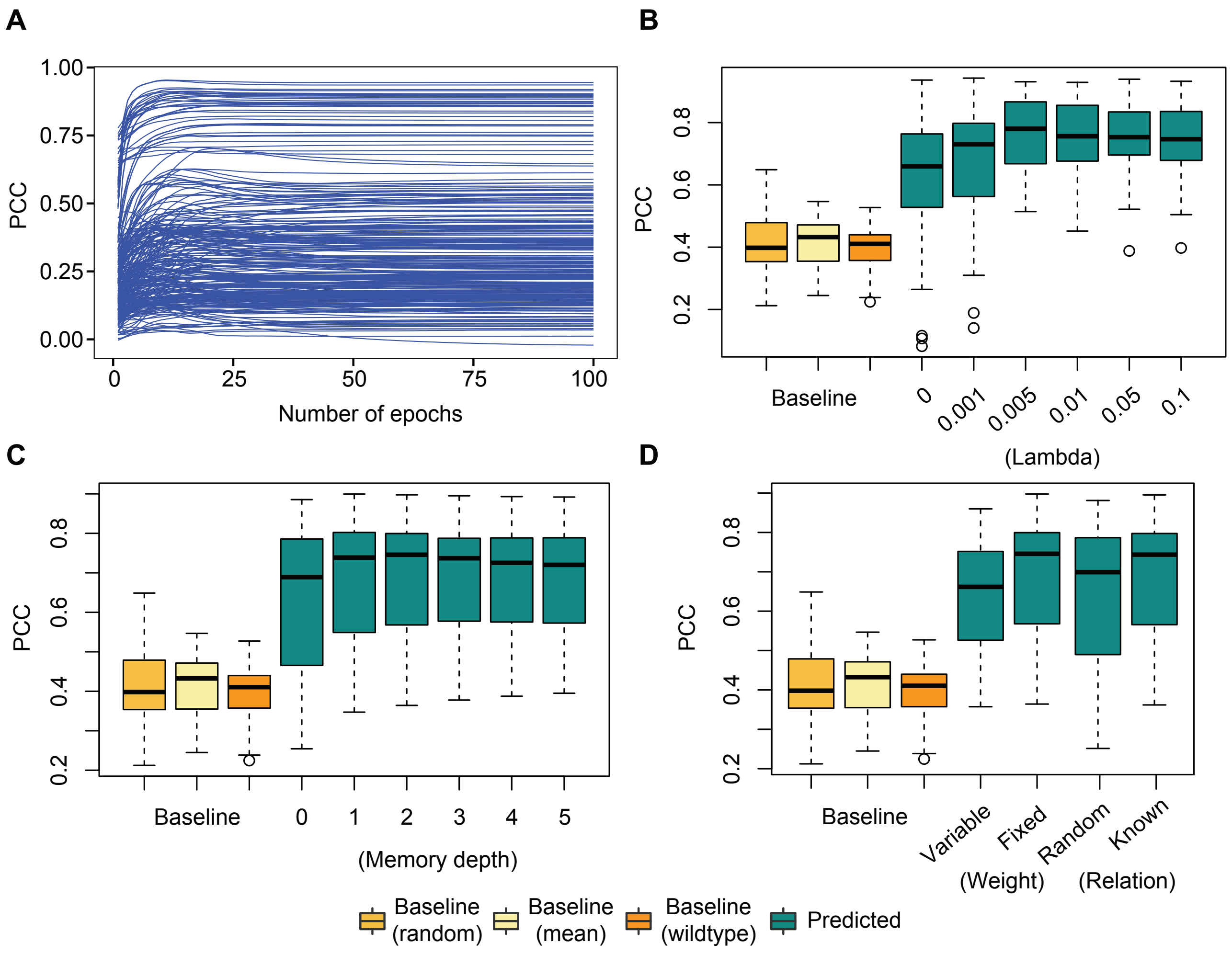

Supplementary Figure 16: Parameter optimizations for RNN in transcriptome layer. (A) number of epochs. (B) regularization parameter. (C) memory depth. (D) effect of i) invariance of weight parameters through time and ii) known relations for connecting nodes.

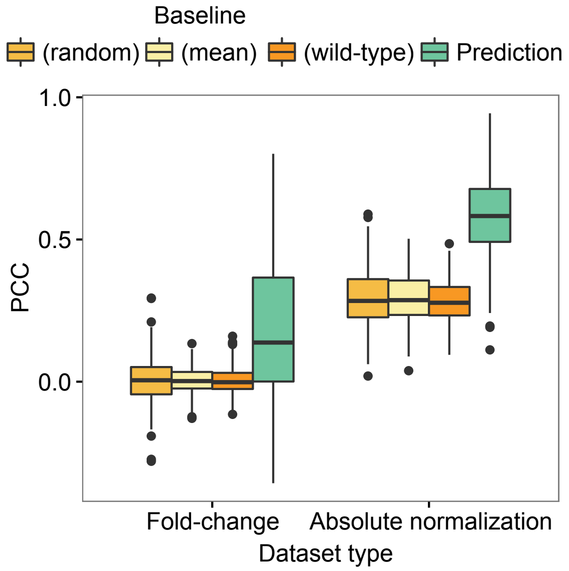

Supplementary Figure 17: Comparison of prediction performance between two different types of datasets. We have performed comparative analysis of prediction performance between two different training datasets; One is based on fold-change and another one is based on absolute normalization. Fold-change data was collected from the compendium COLOMBOS v3 whereas absolute-normalized data was from the compendium Ecomics (this work). We extracted the overlapping conditions between two sources. Absolute-normalized data utilizes expression levels of reference conditions (conditions used for base of perturbation) and perturbation conditions separately unlike the fold-change data that collects only differences in expression levels between perturbation condition and corresponding reference condition. Expression data for reference conditions used for each of the perturbations are added in absolute-scale dataset unlike fold-change dataset. Then the cross-validation experiments of Recurrent Neural Network (RNN) we designed (**Supplementary Text**; **Section 3.3**) were performed based on each of two different sources separately. As for fold-change dataset, by definition of fold-change, it does not have any wild-type expression levels. Thus, unlike the absolute level-based RNN that uses average of expression levels in wild-type conditions (MG1655 with no stresses and no genetic perturbations) as background values of internal nodes, background values of RNN trained on fold-change data were computed by taking average of fold-changes across all perturbations.

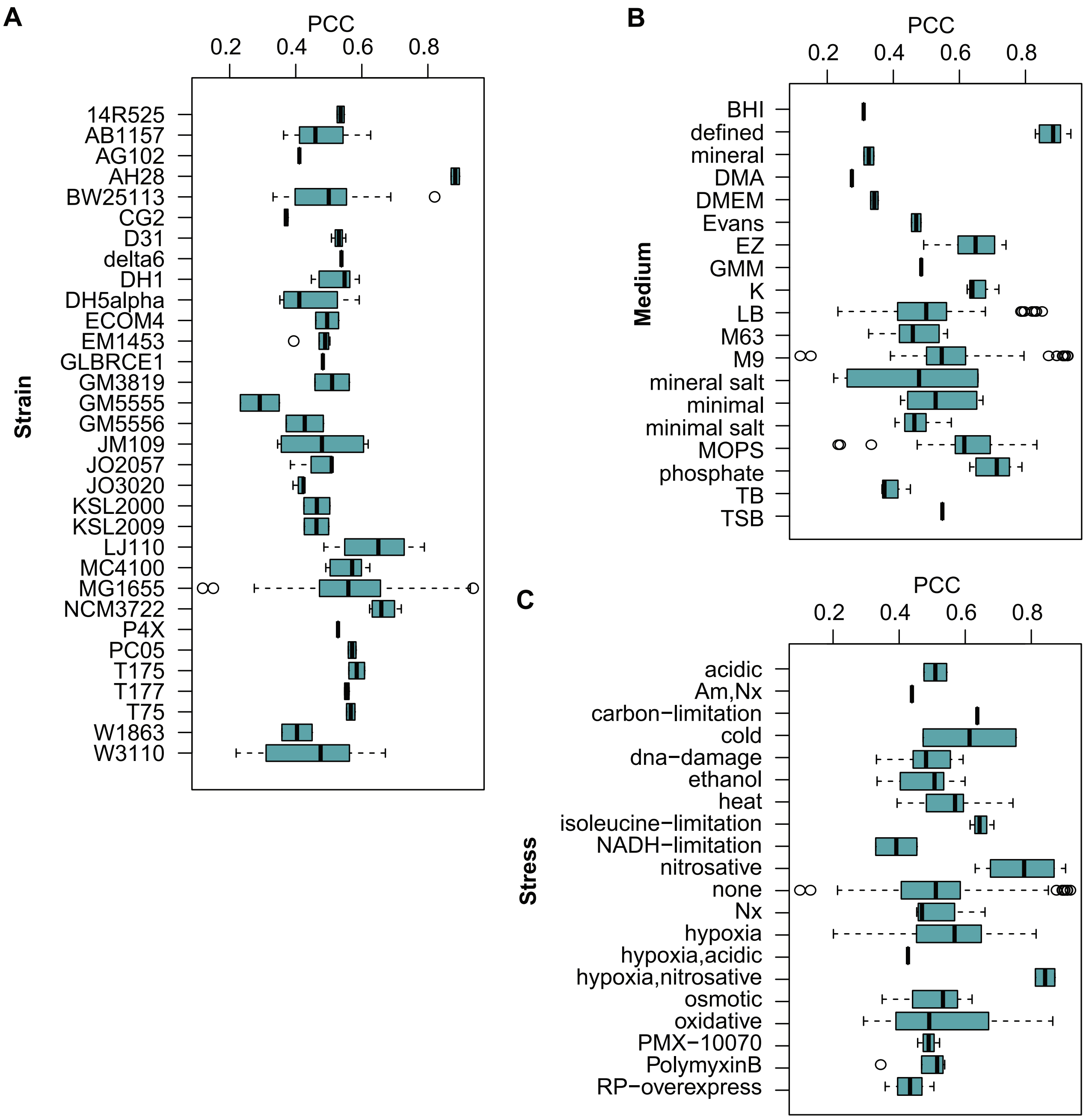

Supplementary Figure 18: Prediction performance of transcriptome layer for different strains (A), different media (B), and different stresses (C). Am, ampicillin; Nx, norfloxacin; RP, recombinant protein

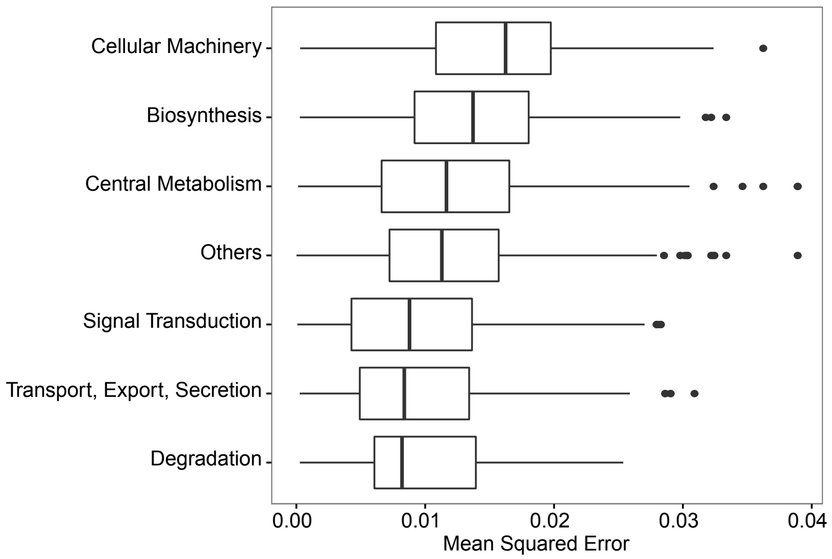

Supplementary Figure 19: Sensitivity analysis of the model parameters. Mean squared error (MSE) between predicted expression levels and known expression levels is computed for each gene across all conditions. MSE of all 4096 genes are grouped into seven pathway groups.

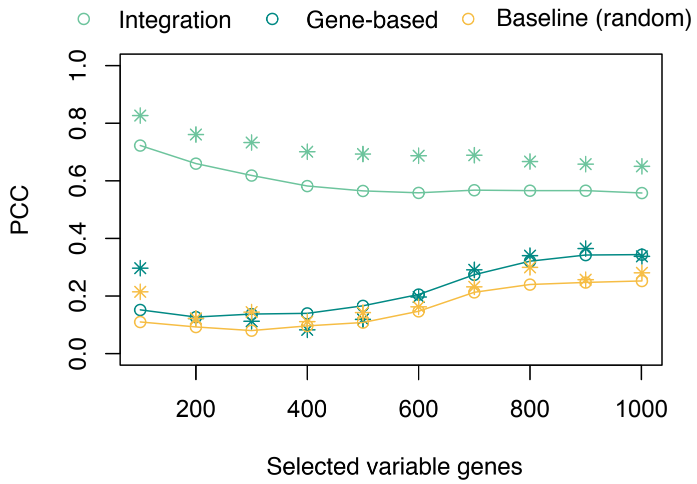

Supplementary Figure 20: Prediction performance for proteome layer and number of selected variable genes. The genes for testing prediction performance are selected based on variability and we increase the number from 50 to the total (1001).

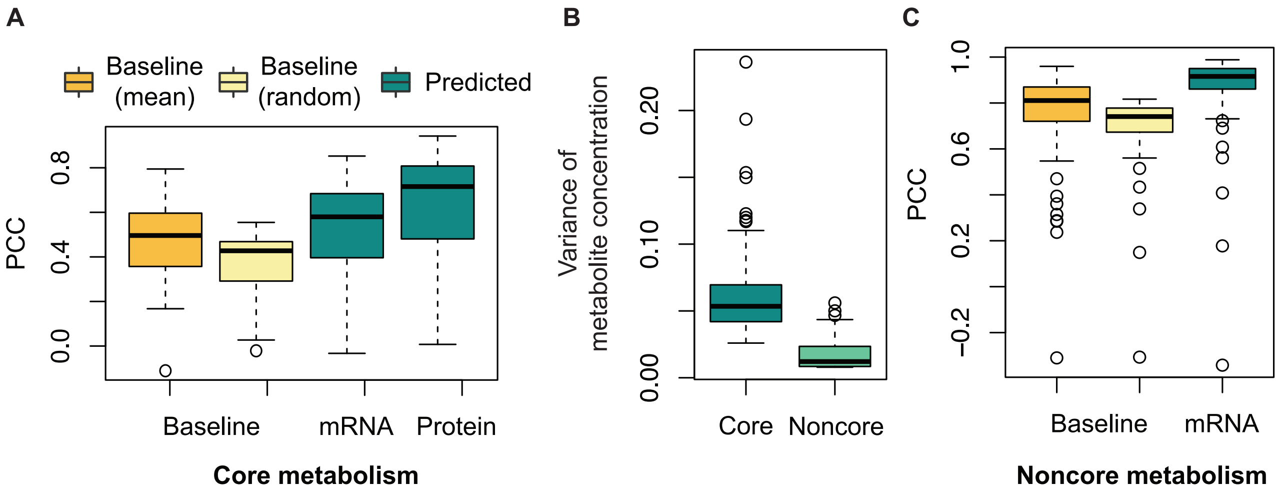

Supplementary Figure 21: (A) Prediction performance of concentrations of 126 metabolites from 75 protein expression levels in core metabolism. (B) Variance of metabolite concentrations in core and non-core metabolism. (C) Prediction performance of 230 metabolites from 4096 gene expression levels in non-core metabolism.

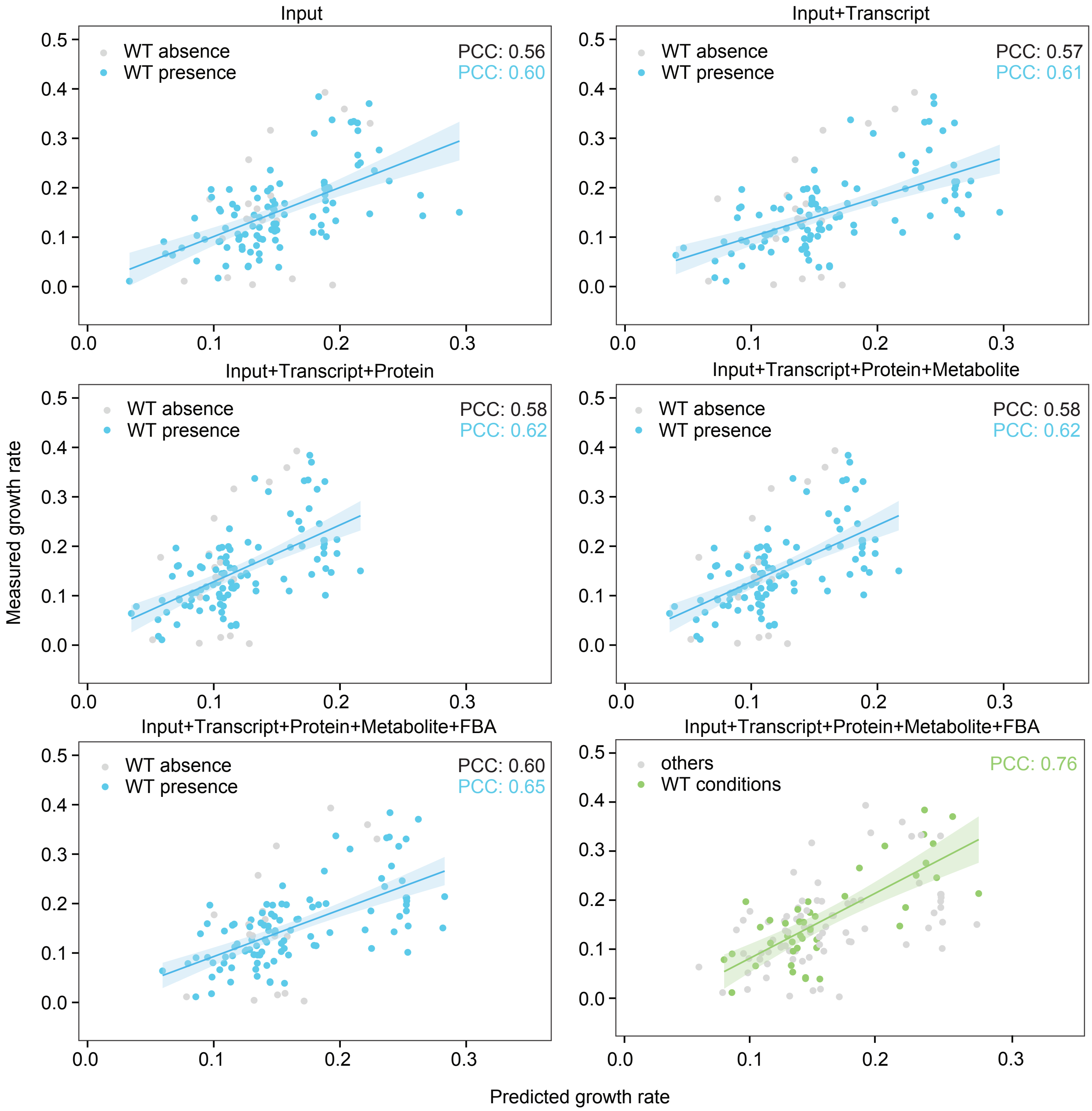

Supplementary Figure 22: Increase in prediction performance for growth rate as each layer is added. The model first uses input features only to predict growth rate and gradually adds the transcriptome, proteome, metabolome, and fluxome layers. PCC was measured between predicted growth rates and measured growth rates from leave-one-condition-out cross validation. 120 conditions were tested for validating prediction of the integrated model. Among them, 101 were cases with wild-type conditions in training set (denoted as WT presence), 60 were novel wild-type conditions (in green).

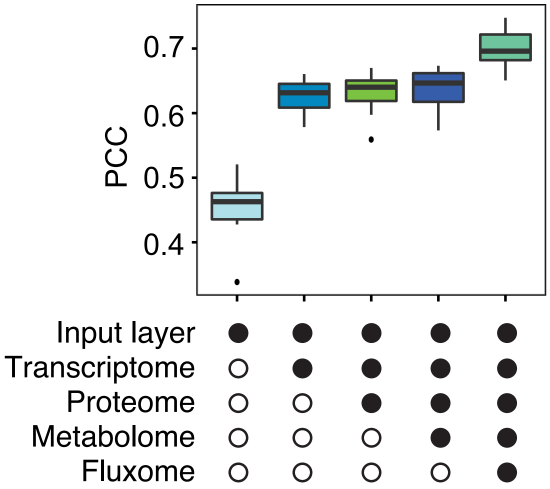

Supplementary Figure 23: 53 conditions showing a distinctive improvement in prediction when adding additional layer from input layer.

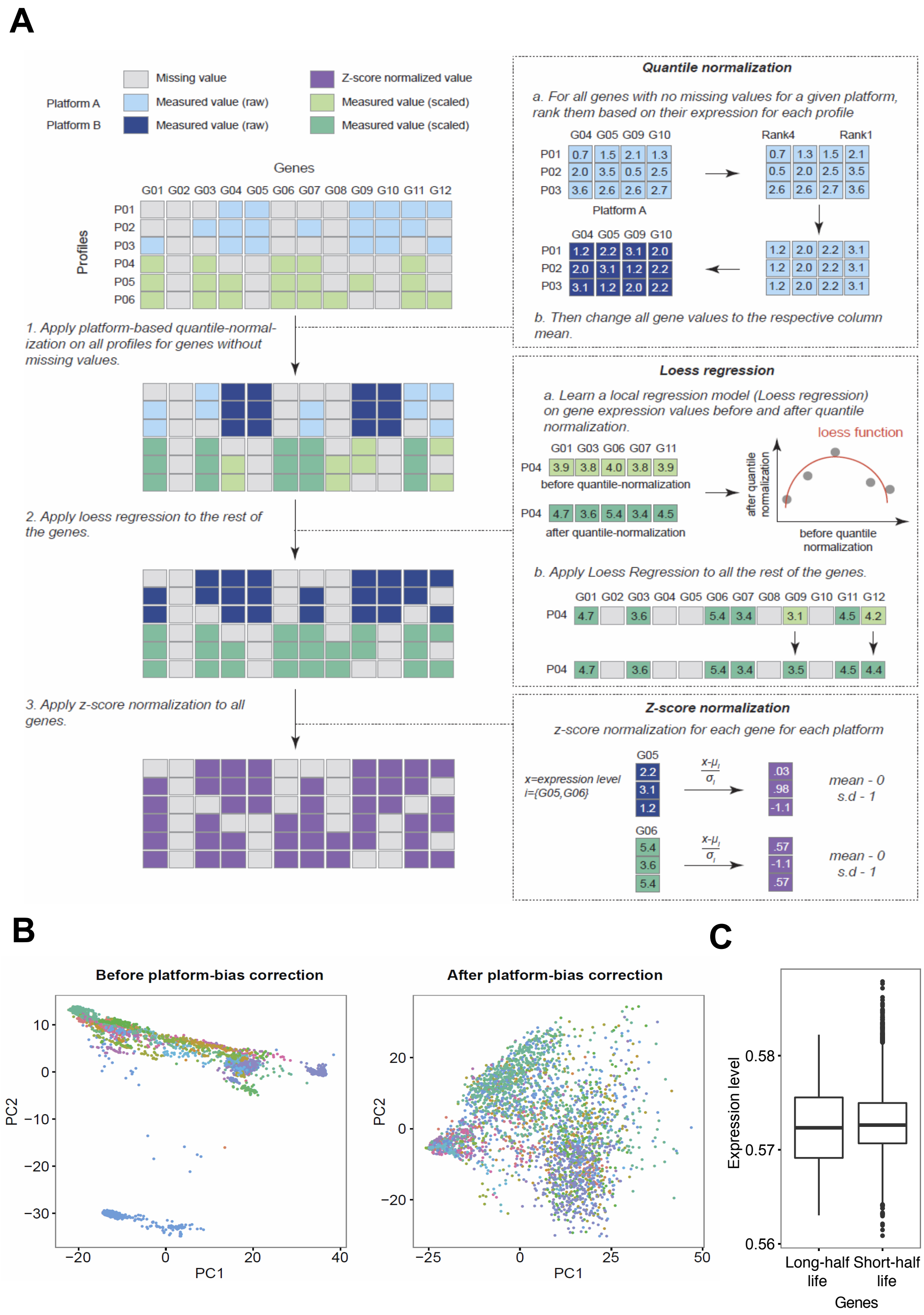

Supplementary Figure 24: Ecomics platform bias correction methodology. (A) Genome-wide profiles are first quantile-normalized within their respective platform. Loess regression is then used to predict missing values. Finally z-score normalization corrects platform bias. (B) Principal Component Analysis depict the distribution of transcriptional profiles in Ecomics before (left) and after (right) platform normalization. Blue, two-channel array; Green, one-channel array; Orange, RNA-Seq. (C) Comparison of normalized expressions between genes with short half-life (0.573 ± 0.004) and genes with long half-life (0.572 ± 0.003). The mean difference was statistically insignificant ($P = 0.41$).

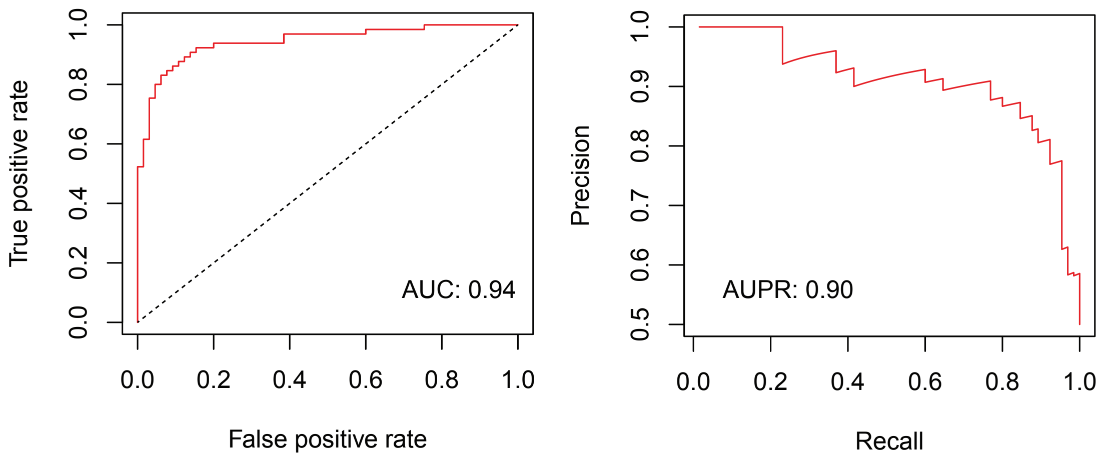

Supplementary Figure 25: ROC curves for prediction of growth phase based on iterative learning.

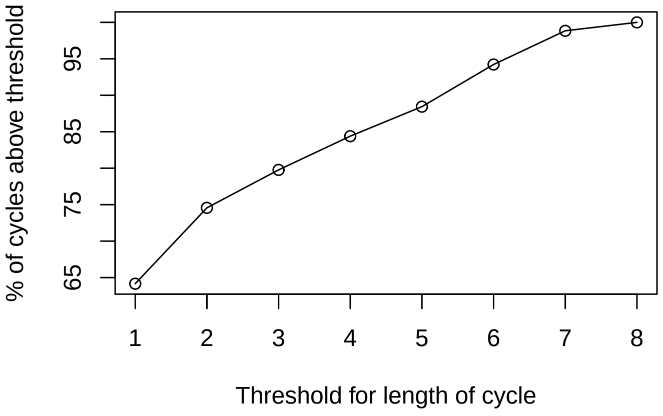

Supplementary Figure 26: Cycles in transcriptional regulatory network in *E. coli*

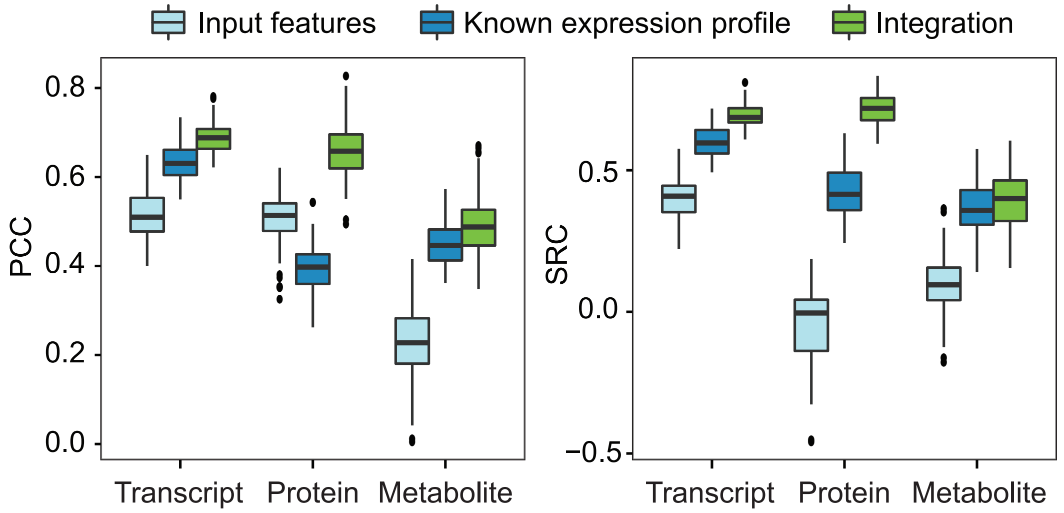

Supplementary Figure 27: Prediction performance of growth rate by three methods of i) using condition characteristics, ii) using expression data, and iii) using both. PCC and SRC were measured between predicted growth rates and measured growth rates from leave-one-condition-out cross validation.

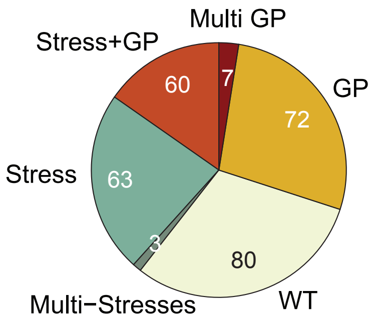

Supplementary Figure 28: Types of testable conditions in the transcriptome data.

2 Supplementary Tables

Phase	Gene
0	*cysG*
0	*dcd*
0	*fadR*
0	*ppk*
0	*cysH*
0	*wzc*
0	*yghD*
0	*fepA*
0	*lacA*
1	*mgtA*
1	*gabT*
1	*sdhC*
1	*putP*
1	*rfbA*
1	*entF*
1	*kefB*
1	*cysA*
1	*trpD*
1	*galE*
1	*mhpD*
1	*fliY*
1	*lplA*
1	*kch*
1	*aspC*
1	*ugpC*

Supplementary Table 1: Genes selected for knock-out experiments. Phase 0 includes 9 genes that were identified as likely candidates in a previous work [1], which we now transcriptionally profiled and added in the compendium. Phase 1 represents the 16 genes that were adaptively selected in this work to maximize GO coverage and their profiles were subsequently added to the compendium.

	Prediction performance		WT baseline
	Same conditions	All conditions	
**10%**	0.56±0.12	0.47±0.15	0.34±0.18
**25%**	0.63±0.07	0.49±0.13	0.34±0.22
**50%**	0.64±0.10	0.52±0.16	0.36±0.20
**75%**	0.67±0.07	0.53±0.13	0.36±0.20
**90%**	0.68±0.06	0.535±0.14	0.35±0.19
**100%**	0.69±0.06	0.54±0.15	0.36±0.22

Supplementary Table 2: Prediction performance and data size. We randomly select 10 times the profiles at the amount of x percentage of the total profiles in the original Ecomics compendium. We run the cross-validation experiments for each dataset. We measure prediction performance for all conditions in the cross-validation experiments as well as one for the same conditions present in all reduced datasets.

Condition	Max growth rate for KO	Max growth rate for WT	% decrease in growth rate
Δ*wcaF*	0.263±0.032	0.279±0.013	5.73%
Δ*mreB*	no growth	0.279±0.013	100%
Δ*yfiP*	0.240±0.029	0.279±0.013	13.97%
Δ*tyrP*	0.249±0.077	0.279±0.013	10.75%
Δ*ycbT*	0.223±0.007	0.279±0.013	20.07%
Δ*gfcC*	0.278±0.025	0.279±0.013	0.35%
Δ*solA*	0.226±0.026	0.279±0.013	18.99%
Δ*fecA*	0.280±0.004	0.279±0.013	0.35% (increase)
Δ*ynfB*	0.238±0.016	0.279±0.013	14.69%

Supplementary Table 3: Validation results of most informative genes for predicting growth rate. We did growth experiments of knockout strains using Keio library for each of informative genes with respect to growth rate prediction (see **Section 4.3** for the experimental details). LB medium was selected not to impose any stresses in nutrients.

Source	# of interactions	# of target genes	Ref
EcoCyc	809	774	[12]
RegulonDB (v8.6)	4,021	3,043	[41]
Cho et al. 2014	6,325	3,134	[69]
Total	8,381	3,948	

Supplementary Table 4: The reconstructed sigma factor network for *E. coli*.

3 Supplementary Methods

3.1 Ecomics: The multi-omics compendium of *Escherichia coli*

3.1.1 Overview

The Ecomics compendium is divided into two major parts, the multi-omics genome-scale profiles and the experimental meta-data (**Section 3.1.2**). In addition to the Ecomics compendium, we have curated genome-scale interaction data for signal transduction, transcriptional, protein and metabolic layers (**Section 3.1.3**). The genome-scale profiles are classified in five groups, based on their omics layer:

- transcriptional (RNA): 3579 profiles of 4096 transcripts from GEO [2], ArrayExpress [3], and SRA [4]
- proteomic: 71 profiles of 1001 proteins from PRIDE [5], ProteomeXchange [6] and various literature sources [7–15]
- metabolic: 696 profiles of 356 unique metabolites from various literature sources. Different synonyms of 356 metabolites profiled in the compendium are consolidated by interrogating from ChemSpider [16], HMDB [17], and PubChem [18]. [11, 19–22, 22–25]
- fluxomics: 43 profiles of 120 fluxes from various literature sources [7, 23, 26–28]
- phenomics: we experimentally measured the growth characteristics (lag, slope, final OD) of 2187 profiles in our lab. In cases where the growth rate is also reported in the published work, that value is also added and annotated accordingly (767 profiles).

For environmental and molecular characterization, we have used several sources in addition to literature curation. We created a chemical composition matrix of 120 attributes to characterize all 112 media used in the Ecomics compendium. Stress definitions were surveyed from the corresponding literature [22, 26, 29–58, 58–77]. For strain characterization, we collected the genetic data from CGSC [78], ecoliwiki [79], EcoCyc [80], and literature, resulting in information for 65 strains with 154 genotypic features. Interaction and network data were also aggregated and used in conjunction with the compendium. The metabolic reaction network of 2,382 reactions of 1,668 metabolites and 1261 genes (iAF1260) was derived from the BiGG [81] database. A protein-protein interaction (PPI) network of 823,098 interactions of 4,250 proteins was constructed by integrating data from bacteriome.org [82], STRING [83], and literature [84–87]. From RegulonDB [88], EcoCyc [80], and other sources [89], we constructed the current state of the *E. coli* transcriptional regulatory network, which consists of three sub-networks: (a) a Sigma factor binding network with 8,381 binding interactions for 7 sigma factors and 3,948 genes, (b) a small RNA regulatory network of 216 binding interactions for 44 sRNAs and 178 genes, and (c) a transcription-factor network of 3809 regulatory interactions for 181 TFs and 1,538 genes. For the signal transduction network, we used our previous work reported in [1], which characterizes 191 events for 105 TFs and 129 metabolites.

3.1.2 Meta-data collection

Each profile in Ecomics is described by several attributes that characterize the environmental and genetic background (**Supplementary Data 6**). These attributes can be grouped in the following categories.

- **Genotype:** We matched strain information from Yale's Coli Genetic Stock Center (`CGSC`) [78] and `Ecoliwiki` [79], as well as information that was present in each publication to assemble the genetic background of each strain used. Mutations at different loci are treated as different alterations for any given gene.
- **Medium:** Similarly, we used information from the respective papers to reconstruct the chemical composition of all media used. Whenever a standard medium was used but no further information is given, we referred to `EcoCyc` [80] to estimate its formulation. In rare occasions, we had to generalize on media compositions (for example for media with casamino acids we assume that all 20 amino acids are present).
- **Growth dynamics:** Growth rate is reported from the curated publication for 767 out of the 4,345 profiles. For filling missing phenotypes in the compendium, we independently measured growth for another 1992 profiles (out of 3,579; 55.6% of all profiles) with transcriptional information, corresponding to 179 conditions (out of 596; 30.0% of all conditions). Similarly, we measured the growth dynamics and covered 80.2%, 82.9% of profiles with proteomics and metabolomics/fluxomics information, respectively. In all cases, measurements include lag time, slope and final OD, as defined in **Section 3.4.3**.
- **Stress:** Stress is defined as any experimental condition that is known to (or is expected to) induce stress-response mechanisms. There is not a clear ontology of stresses, with a notable effort by the Plant Stress Ontology (PSO) project that is currently under development [90]. Here, we introduce a organism-centric approach to draft an ontology in **Section 3.2.4**.
- **Time:** Elapsed time after the strain was exposed to a perturbation and the collection of samples for profiling.
- **Genetic perturbation:** The type of genetic perturbation, if one is present. Values include Wild-type (WT), knock-out (KO), over-expression (OE), mutation (defined as changing one or more nucleotides in the CDS), insertion (an insertion of one or more genes), large-scale deletion (that affects more than one gene).
- **Number of perturbed genes:** Genes involved in perturbations.
- **Temperature:** Temperature in which the experiment was conducted. In cases where temperature is not reported, 37°C is assumed.
- **pH level:** The pH level in which the experiment was conducted. In cases where pH level is not reported, a pH 7.0 is assumed.
- **Compounds:** Any compounds that were added beyond what is expected to be in the respective media. Usually includes chemicals that induce stress (e.g. mutagens).
- **Compound concentrations:** Defined in g/L, Moles or percentages.

3.1.3 Molecular interaction data

Transcription Factor network Transcription Factor (TF) binding information was provided by `RegulonDB` (v8.6) [88]. All interactions with strong or weak experimental evidence (defined as having at least one independent sources of validation) were used, which results in 3,489 interactions of 179 TFs and 1,478 genes. In addition, we added recent datasets to this set [91], resulting to 3,809 TF-gene bindings of 181 TFs and 1,538 genes (**Supplementary Data 5**).

Small RNA network Small RNAs (sRNAs) are non-coding RNAs having 50-250 nucleotides in bacteria. sRNAs can bind either to a protein to modify the function of the protein or to mRNA to regulate its expression level. Numerous studies report that many sRNAs are involved in stress response regulation [92–100]. To reconstruct the global map of sRNA-mediated regulation for *E. coli.* we downloaded known regulations from `RegulonDB` (v8.6) [88] and `EcoCyc` [80], which results in 216 experimentally validated sRNAs interactions of 44 sRNAs and 178 genes (**Supplementary Data 5**).

Sigma factor network Sigma factors are transcription initiation factors that enable a specific binding of RNA polymerase to gene promoters. Cell utilizes different sigma factors that bind to a gene promoter, which in turn modulates transcriptional activity of the target gene in order to react to different environmental signals [101–103]. We built a global map between 7 sigma factors and target genes by compiling known information from multiple sources [80, 88, 89]. The consolidated network is composed of 8,381 interactions between seven sigma factors and 3948 genes (**Supplementary Table 1**, **Supplementary Data 5**).

Signal transduction network For the signal transduction network, we used our previous work reported in [1], which consists of 191 interactions for 105 TFs and 129 metabolites.

Protein-protein interaction (PPI) network A protein-protein interaction (PPI) network maps physical interactions between proteins [84]. We compiled the interaction data from four distinctive sources. We collected data from (a) affinity purification approaches followed by mass spectrometry (AP/MS), a total 11,496 interactions among 1,631 proteins [85, 86], (b) binary-Y2H experiments, a total of 3,936 interactions among 2,045 proteins [87], (c) the `STRING` public database, a total of 817,650 interactions among 4,147 proteins [83], (d) the `EcoCyc` database, a total of 701 interactions [80]. Ultimately, this led to a reconstructed *E. coli* PPI network of 823,098 interactions for 4,250 proteins, which is the most comprehensive PPI network for the organism until now [104] (**Supplementary Data 5**).

3.1.4 Strain and Medium representation

Strains are characterized with 154 genotypic features that correspond to DNA variations (i.e. deletions, insertions, polymorphisms) with respect to the ancestral K-12 strain (**Supplementary Fig. 1**). Similarly, the media are characterized by 120 chemical features (**Supplementary Fig. 2**). There are 65 strains and 112 media in Ecomics.

3.2 Analysis of the Ecomics compendium

3.2.1 Overview

Ecomics includes information for 65 strains, 112 media, 52 stressors and 286 genetic perturbations. Its 4,346 genome-wide profiles span the transcriptional (3,579 profiles), protein (71 profiles) and metabolic (696 profiles) layers. A "condition" is defined a combination of the genetic background (strain, genetic perturbations) and the environmental setting (medium, stress). The compendium retains 649 different conditions (596, 36 and 53 conditions with transcriptome, proteome and metabolome profiles, respectively, with 68.2% of them with 3 or more profiles (replicates from one or more studies). 65.6% of all profiles in the compendium include either a stressor or a genetic perturbation and among them 517 profiles have them both (**Supplementary Fig. 4A**). As in **Supplementary Fig. 4B**, the condition with most profiles (187 profiles; 4.2% of Ecomics) is the MG1655 strain in MOPS medium with carbon-shift from glucose to lactose.

3.2.2 Strain

The strains with the most profiles in Ecomics is MG1655 (64.4%), BW25113 (9.6%) and W3110 (7.8%), with profile distribution depicted in **Supplementary Fig. 5**). Only the MG1655 and BW25113 strains had profiles in all four layers. Genotypic map of the strains used (**Supplementary Fig. 1**) are characterized with 154 genotypic features show sparsity in shared features, where the number of genotypes for each strain ranges from 1 to 11. Although there are no genotypic features that are prevalent in all strains, many of them are locally shared between 3 or 5 strains (e.g. knockouts in the *crl* genes are shared in 5 out of the 65 strains).

Expression-based strain ontology. We used average linkage hierarchical clustering (UPGMA) [105] to construct both genotype-based and expression-based ontologies for Ecomics strains. Profiles of MG1655 strains in LB medium with no stress or genetic perturbations were used. Expression profiles in a given condition were averaged for each gene to have a representative expression profile for the condition. Then the expression level of each gene was scaled between 0 and 1 by min-max normalization [106], which is also discussed in **Section 3.3.3**. The distance between a pair of vectors, both in the case of expression levels and phenotype features was measured by PCC. **Supplementary Fig. 9A** depicts the ontology that is generated based on the phenotypic characteristics. **Supplementary Fig. 9B** illustrates the ontology that results from the integration of these two sources of information by using linear superposition with equal weights. Interestingly, the cophentic correlation of the genetic and expression-based ontology is 0.21. The same distance between the integrated ontology and either of the two sources (genetic, expression-based) is 0.52. Thus, relying solely on genetic characteristics to infer genome-scale expression and phenotypic traits can clearly be misleading.

Correlation between expression profiles and genetic features. To investigate the correlation of shared genotypic characteristics to the resulting expression profiles, we identified the conditions where two or more strains had expression profiles and calculated the strain pairwise correlation, thus creating a expression-based covariance matrix, where row/columns are the 65 strains. We then constructed a genotype-based covariance matrix and compare the two based on Pearson's correlation between two vectorized lists of matrices (0.58).

3.2.3 Medium

In the 112 media in Ecomics, glucose and glycerol are the most popular carbon source. M9 [107], LB [108], and MOPS [109] are the three most abundant media (**Supplementary Fig. 10**. the number of chemicals used for each medium ranges from 4 to 45 (15.3±9.0) (**Supplementary Fig. 2**. Similarly to the strain ontology, we created media ontologies based on the genome-scale expression levels (**Supplementary Fig. 10B**). We applied the same method as described in strain ontology analysis (**Section 3.2.2**). All profiles of the MG1655 strain (largest number of profiles) with no stress and genetic perturbations were used for this test.

3.2.4 Stress

Conditions in Ecomics include 52 stressors (**Supplementary Fig. 7A**) and some of their combinations, divided in 16 groups (**Supplementary Fig. 7B**). The most abundant stressors were nutrient-shift (359 profiles), followed by oxygen starvation (280 profiles). Among the 5 different types of nutrient-shift, glucose to lactose shift was the most dominant diauxie, followed by glucose to acetate shift. There were 15 different antibiotics in the compendium, with Norfloxacin having the most profiles (200 profiles). We created an expression-based stress ontology which provides an insight on what conditions result to similar genome-scale expression in the bacteria that are exposed to them (**Supplementary Fig. 11**).

3.2.5 Genetic Perturbations

The 286 genetic perturbations (e.g. 183 knockouts, 63.9%; 91 over-expressions 31.8%) cover 27% of KEGG pathways, 44% of GO Biological Processes (BP), 7% of GO Molecular Functions (MF). A pathway, process or molecular function is covered if the compendium has one or more profiles for at least one of its genes.

3.2.6 Targeted experimentation to increase GO coverage.

To increase the GO coverage of Ecomics, we investigated which new conditions would lead to the most informative transcriptional profiles. We first transcriptionally profiled and included in Ecomics 9 KO experiments that we had identified before [1] as being highly informative for both GO coverage and model performance (**Supplementary Table 1**). We assumed that a GO term is represented in the compendium if the compendium has profiles where one or more genes that include that GO term have been perturbed. With that assumption, **Supplementary Fig. 8A** depicts the increase in coverage after the inclusion of transcriptional profiles that correspond to novel gene perturbations in the compendium. We identified the gene rank iteratively under the assumption that all genes of higher rank have been perturbed. Based on the resulting ranked list, we performed transcriptional profiling for the top 16 genetic perturbations (in triplicate; 48 profiles total, **Section 3.4.1**), which led to an increase in coverage by 19.8% for KEGG, 24.3% for BP and 63.9% for MF (**Supplementary Fig. 8B**, **Supplementary Data 1**). The list of 16 genes for knock-out experiments are in **Supplementary Table 1** (phase 1). We also performed differential expression analysis between pre-processed gene expression profiles for each KO gene of BW25113 strain using `DESeq2` [110] to find novel genes that are affected by genes we perturbed.

3.2.7 Variability analysis.

We identified *secD*, *galE*, *mcrB*, *phoR*, Δ(*ara-leu*)7697, Δ(*codB-lacI*)3 mutations as the key factors in strain-dependent variability (**Supplementary Fig. 5**). For *galE*, it has been known that when a *galE* mutant is grown in the presence of D-galactose, growth is arrested due to low availability of CTP and UTP, which results in reduced RNA synthesis [111]. Moreover, PhoR has been known to indirectly sense and to respond to variations in the level of extracellular inorganic phosphate, which is one of essential macronutrients for biological growth [112]. Hence, dysfunction of the phosphate regulatory mechanism by mutating *phoR* is expected to impact global changes in the cell, which is on a par with the medium variability analysis results, where phosphate-containing compounds are a key correlate to expression variability.

Focusing on media (**Supplementary Fig. 6B**), our analysis revealed that Na2H(PO4), H3BO4, KH(PO4), Fe(III)citrate, yeast extract, MgSO4, NH4Cl, sodium citrate, H3BO3, biotin and FeSO4 are key factors of expression variability. Na2H(PO4) and KH(PO4) are the sole source of phosphate in most defined media and any perturbation in the phosphate availability will evoke a global response [113,114]. MgSO4 and NH4Cl are sources of magnesium and nitrogen that are critical for key enzymes [113] and synthesis of amino acids [114]. Yeast extract is an undefined media so the observed high variance in expression is expected.

Similarly, transcriptional responses within replicates exhibited stress-dependent variability (**Supplementary Fig. 12**). Expression variability is measured as the coefficient of variation for genome-scale gene expression, normalized for the same strain and medium (MG1655 and M9 medium, respectively). We found that environmental conditions that trigger targeted specific biological processes have lower variance in expression within replicates than environments that target multiple/global biological processes. We confirmed that the observed expression variability is not an artefact of laboratory biases or sample size: the Pearson Correlation Coefficient (PCC) between the expression variability and the number experimental laboratories or publications is -0.09; similarly, the PCC between the expression variability and the number of replicates per strain is -0.11. Antibiotics such as norfloxacin (Nx), ampicillin (Am), and Polymyxin B that act on specific biological processes including inhibition of cell division and altering cell wall, lead to profiles with lower variability among replicates, compared to heat and acidic stress that are associated with a global transcriptional response (0.18 ± 0.07 vs. 0.34 ± 0.15 coefficient of variation, respectively; **Supplementary Fig. 10**). In addition, acidic stress led to higher variance than heat stress (0.46 ± 0.11 vs. 0.22 ± 0.08 coefficient of variation, respectively). This result argues that *E. coli*'s heat response program is more robust than acid homeostasis. Indeed, *E. coli* has a well established system to up-regulate heat responsive genes through σ32, and to protect protein aggregation and misfolding by expressing several chaperons [115]. In contrast, to cope with acidic stress, *E. coli* has three response systems: a σ38-dependent, a glutamate-dependent and an arginine-dependent response [116]. However, none of these systems induces the expression of chaperones as it is evident from our transcriptional profiling analysis and reported in literature [117], which is expected to lead in higher protein instability. Similarly, the high expression of chaperon proteins in hypoxic conditions [118] may also play a role on the low expression variance within hypoxic replicates. The observed variance in our profiles also holds for combinations of stresses. For instance, in the case of simultaneous treatment with acidic and hypoxic stress, the transcriptional variability is the mean of that of the respective stresses. High variability in recombinant protein stress is expected, as different recombinant proteins and expression systems impose different metabolic burden to the cells [119].

3.3 Data-driven, genome-scale predictive model

3.3.1 Overview

We built a genome-scale model that aims to predict an output vector y that contains the expression levels of mRNAs, proteins, metabolites, metabolic fluxes and growth dynamics, for a given experimental condition. The experimental condition is represented as an input vector x with features that contain information about the genetic (strain, genetic perturbation) and environmental (medium, stress) background. The model was designed to be modular, in order to allow for a better representation of biological organization, an easier manipulation of individual modules and to avoid dimensionality issues. We evaluated various statistical techniques in their ability to capture biological structure and make accurate predictions. The final model includes Recurrent Neural Networks (RNN; [120]) with regularization of sigmoid activation functions [121] for the transcriptome layer and LASSO constraint regression [122] for the other layers (using R package `glmnet` [123]). The model is trained on the Ecomics compendium and all the network resources that are summarized in **Section 3.1.1**. Our analysis is focused on profiles for samples in the exponential phase, as the amount of data is sufficient for a rigorous assessment of the model.

The input features (x) are directly used into transcriptome layer to predict genome-wide expression levels of genes. Then the rest of layers are predicted from the transcriptome layer. The motivation of this approach is that the profiles from a wide spectrum of environmental conditions are mostly enriched in transcriptome layer in the compendium. The responses in the proteome are predicted from the transcriptome layer. The metabolome layers are predicted from the transcriptome layer as well as the proteome layer. Once complete, the genome-scale fluxes are predicted based on the constraint-based model by imposing the constraints of fluxes from the three layers of input, transcriptome, and proteome. Finally, growth rate is predicted by consensus of predictions made from all five layers (input, transcriptome, proteome, metabolome, fluxome).

3.3.2 Sample representation

A sample *i* is defined as the pair of multi-dimensional vectors $(\mathbf{x}^{(i)}, \mathbf{y}^{(i)})$, where $\mathbf{x}$ can be thought as the ***input*** or the independent variables of the model and $\mathbf{y}$ can be thought as the ***output***, or the dependent (response) variables of the model. The superscript denotes the index of the sample and here a sample is a profile from the Ecomics database. The 612-by-1 vector x contains features that encode information about the environmental condition and genetic background, hence we will refer to them as the ***conditions*** of the sample. The $\mathbf{y}$ vector consists of two parts: a 7,835-by-1 vector of variables that encode for the organism's biomolecular abundances and metabolic fluxes and a vector of variables that correspond to specific phenotypic traits. In our formulation, we assume that we have only one phenotype that we want to predict, namely the growth rate. As such, the size of the output vector $\mathbf{y}$ is 7,836-by-1.

More specifically, the input vector $\mathbf{x}$ consists of four types of features:

- **Strain information**: the strain information is represented as 154 genotypic features $\{x_i\}_{i=1}^{154}$ where each feature x_i is a discrete random variable and it denotes a DNA-level status of a gene (e.g. $x_2 = \{0, 1\}$ where x_2 can represent the *rfb* gene, the values 0 and 1 denote wild-type and *rfb* knockout, respectively).
- **Medium**: the medium composition is expressed as 120 chemical features $\{x_i\}_{i=155}^{274}$ where each feature x_i is a ordinary random variable and it denotes the availability of either a defined chemical feature

(e.g. $x_{122} = \{0, 1\}$ represents K_2HPO_4, 0 and 1 denotes absence and presence of the chemical, respectively) or an undefined chemical feature (e.g. yeast extract).

- **Environmental abiotic stress**: the environmental cues are expressed as a 52-by-1 vector $\{x_i\}_{i=275}^{326}$ where each is a binary random variable and represents a stress (e.g. $x_{276} = \{0, 1\}$ represents heat-shock stress, 0 and 1 denotes absence and presence of the stress, respectively).
- **Genetic perturbations**: Genetic perturbations are a 286-by-1 vector $\{x_i\}_{i=327}^{612}$ where each x_i is a binary variable denoting the presence or absence of a genetic perturbation (a perturbation includes knock-out, mutation, over-expression, and insertion). For example $x_{342} = \{0, 1\}$ corresponds to the *rpoS* gene having an original copy (0) of the gene or being knocked-out (1).

The output vector $\mathbf{y}$ consists of five types of features:

- **Transcriptome**: represented with a 4,096 element vector $\{y_i\}_{i=1}^{4096}$ where each feature y_i is a continuous variable and it denotes the absolute mRNA expression level of a gene.
- **Proteome**: represented with a 1001 element vector $\{y_i\}_{i=4097}^{5097}$ where each feature y_i is a continuous variable and it denotes an absolute expression level of a protein.
- **Metabolome**: represented as a 356 element vector $\{y_i\}_{i=5098}^{5453}$ where each feature y_i is a continuous variable and it denotes an absolute expression level of a metabolite.
- **Fluxome**:represented as a 2382 element vector $\{y_i\}_{i=5454}^{7835}$ where each feature y_i is a continuous variable and it denotes a relative flux of a reaction.
- **Cellular phenotype**: The growth rate is represented by a scalar in the last position of the y-vector, (y_{7836}), as a continuous variable with (h^{-1}), which is a continuous random variable.

3.3.3 Transcriptome module

For predicting transcriptomic response from input x, we employ a Recurrent Neural Network (RNN) framework. A RNN is a special type of an Artificial Neural Network that allows feedback and hence it is capable to encode for memory or past internal states [124–127]. This enables it to model biologically relevant dynamic behaviors such as feedback-loop of molecular interactions in a cell.

To simulate interactions of extracellular factors and biomolecules in a cell (e.g signal transduction pathways) as well as interactions between intracellular biomolecules (e.g. transcriptional regulation), the architecture of RNN directly connects input nodes into output nodes and allows connections between output nodes. In other words, we avoid to use hidden nodes in the RNN architecture. The representation allows better interpretation of the constructed model as well as integration of known biological evidences, which potentially helps to avoid over-fitting. The schematic diagram of RNN representation that predicts transcriptomic response from input features is depicted in **Supplementary Fig. 13**.

Data preparation We focused our analysis for profiles in the exponential phase, where adequate data exist for model evaluation. We first extracted the transcriptome profiles that correspond to samples in the exponential phase from the compendium (2610 profiles). We then used min-max standardization on the absolute scale values for each gene, to be able to compare and make the training process easier to initialize and converge [106]. The standardization process was applied for each entry i of the first 4,096 entries in the output variable y, which correspond to the transcriptome layer. The variable values were then updated after standardization:

$$y_i^{standardized} = \frac{y_i - min(y_i)}{max(y_i) - min(y_i)} \tag{1}$$

Parameters and activation function **Connection weights.** Each connection $c_{i,j}$ between a node i and a node j has a weight $w_{i,j}$ associated with it (**Supplementary Fig. 13**). We represent the weights for connections between input nodes x (612 nodes) and output nodes y (4096 nodes) as w_x and the weights for connection among output nodes y as w_y, so that the set $W = \{w_x, w_y\}$ includes all weights of the RNN. Weights are initialized based on a normal distribution $\mathcal{N}(0, 0.05^2)$, which provides a balanced weight range and lead to fast convergence for the Ecomics dataset.

Activation function. The sigmoid function ($h(x) = \frac{1}{1+e^{-x}}$) was used as the activation function for each node of the RNN, as it sufficiently approximates the Hill function dynamics of regulatory interactions and restricts the output between 0 and 1. In addition, we performed n iterations (updates) on calculating the output, where n is the memory depth and capture cycles and feedback loops that might be present in the RNN. The initial state of the output nodes $y^{(0)}$ (i.e. gene expression value) was set to the average gene expression levels in the training data under WT conditions (MG1655 with LB or M9 medium with no stresses and no genetic perturbations). As such, given input x and weights $\{w_x, w_y\}$, the output vector y is iteratively computed by:

$$y^{(i)} = h(w_x x + w_y y^{(i-1)}) \tag{2}$$

for $1 \leq i \leq n$, where $y^{(i)}$ is the state of the output vector y at iteration time i.

Training weights

Objective. During the training phase, the goal is to find the RNN connection weights so that the RNN has the best representation of the training data, i.e. its output $y^{(n)}$ approximates well the target output $\hat{y}$ (gene expression for all genes in the training dataset D). This objective can be represented as minimization of the residual sum of squares, or cost function $C_o(w)$ between $y^{(n)}$ and $\hat{y}$ for D:

$$C_0(w) = \frac{1}{2|D|}\frac{1}{M}\sum_{y}^{Y}\sum_{i=1}^{M}(\hat{y}_i - y_i^{(n)})^2 \tag{3}$$

where $|D|$ is the number of conditions in the training dataset (262 conditions) and M is the number of genes in a single profile/condition (4,096 genes).

Back-propagation through time (BPTT). We use BPTT [128] to train the weights W in our RNN model. In BPTT, the RNN is unfolded to interconnected feed-forward neural networks (FFNN), with the node values at each FFNN representing the state of the output at a given time (iteration), as shown in **Supplementary Fig. 13**. Once the RNN is unfolded to a chain of FFNNs, standard back-propagation is used to train the weights of the network [129]. Stochastic gradient descent [130] is used to update the weights through this procedure, with the following update rule:

$$w_{i,j} \leftarrow w_{i,j} - \alpha\frac{\delta C_0^y}{\delta w_{i,j}} \text{ for all } y \epsilon D \tag{4}$$

With α being the update or learning rate (set at 0.01). Note that the update is performed for several iterations, or epochs, which are determined empirically (see next section).

Parameter optimization We evaluated the effect of various hyper-parameter in the performance of RNN and selected the optimal values for the number of epochs, memory depth, type of relations between features, dimensionality reduction methods and the regularization techniques. More specifically:

Number of epochs. An epoch is defined as one training iteration over all samples in the training dataset. We performed a Leave-one-condition-out (LOCO) cross-validation (**Section 3.3.3**) on Ecomics for the transcriptional profiles of 178 transcription factors in the exponential phase. We opted on using only this dataset instead of all genes in Ecomics due to computational constraints. Since TFs are the most representative genes for the enacted regulatory programs in any given conditions, this set encapsulates well the genome-wide changes for each condition. From the 493 conditions with transcriptome profiles available (exponential phase) in Ecomics, 262 of them can be used as part of a LOCO cross-validation, as when excluded from the training dataset, there is at least one condition that has the same value for one of more features of the genetic background or environmental setting. In this set of 262 conditions, we evaluated the effect of the number of epochs, as shown in **Supplementary Fig. 16A**. We found that in 63% of the conditions, 50 epochs were sufficient to achieve convergence (i.e. $\Delta PCC \leq 0.01$), while in all cases, convergence is achieved within 100 epochs. Therefore, we set the number of epochs to 100.

Regularization. To avoid over-fitting and create a sparse representation of the transcriptional organization, we used L_1 regularization (weight decay) [131] to the original cost function:

$$C = C_0 + \frac{\lambda}{n} \sum_{i,j} |w_{i,j}|$$

where C_0 is the original cost (the equation 3), n is the size of the training set and λ is the parameter for adjusting the relative importance of the regularization term compared to C_0. Then taking the partial derivatives of the cost function with respect to w yields

$$\begin{cases} \dfrac{\theta C}{\delta w_{i,j}} = \dfrac{\delta C_0}{\delta w_{i,j}} & \text{if } w_{i,j} \text{ is bias} \\ \dfrac{\theta C}{\delta w_{i,j}} = \dfrac{\delta C_0}{\delta w_{i,j}} + \dfrac{\lambda}{n} sign(w_{i,j}) & \text{otherwise} \end{cases}$$

The biases in w don't change from the regularization term and thus only non bias weights are updated from the equation (6) as

$$\begin{cases} w_{i,j} \leftarrow w_{i,j} - \alpha \dfrac{\delta C_0}{\delta w_{i,j}} & \text{if } w_{i,j} \text{ is bias} \\ w_{i,j} \leftarrow w_{i,j} - \alpha \dfrac{\delta C_0}{\delta w_{i,j}} - \dfrac{\alpha\lambda}{n} sign(w) & \text{otherwise} \end{cases}$$

λ is chosen based on RNN performance observed from LOCO cross-validation of the data set of 178 transcription factors (**Supplementary Fig. 16B**). Our results show that the optimal performance (PCC= 0.76±0.12) is achieved when λ approximates the value of 0.005, which was selected for all subsequent experiments.

Fixed or time-variant weights. We have considered the case where weights are changing through time unfolding, to capture different correlations that might be present in feedback loops. We performed LOCO cross-validation (described in **Section 3.3.3**) from the transcriptome data of 2,610 profiles of 178 transcription factors for two RNN models: one where weights are variable through time/iteration and another with fixed weights (**Supplementary Fig. 16D**). Weights are fixed by averaging weights through time for any given connection. Our analysis shows that fixed weights are faster to train and lead to equal or better ANN predictors (PCC=0.68$\pm$0.14) than variable weights (PCC=0.64$\pm$0.17).

Integration of biological knowledge to network topology. We have evaluated whether the network interaction data that has been experimentally validated confer extra information to the predictive model. We compared the performance of RNNs with and without prior knowledge of the transcriptional regulatory network (**Sections 3.1.3, 3.1.3, 3.1.3**). Performance comparison was conducted as described in **Section 3.3.3**. Our results show that the RNN with a priori connections (PCC 0.68$\pm$0.14) is better to the one without it (PCC=0.63$\pm$0.20).

Memory depth. To get a sense on what memory size (n) will capture the majority of feedback loops in *E. coli*, we first investigated the transcriptional regulatory network (TRN) compiled from public repositories (e.g. `RegulonDB`) and literature for *E. coli* (**Section 3.1.3**). In total, 173 cycles were detected, ranging from one cycle (self-loop) to length of 8 (**Supplementary Fig. 26**). 65% of cycles were self-loops and 85% of all cycles are below a cycle length of 4. Next, we trained RNN with different memory sizes and assessed the effect of memory on the RNN performance. As shown in **Supplementary Fig. 16C**, a memory depth of 2 is sufficient for our purposes, which captures 75% of known cycles.

Model evaluation

Leave-one-condition-out (LOCO) cross-validation. For model validation, we leave out profiles of a condition from the dataset and build a model from the rest and prediction performance of the model is tested from the condition left-out. This procedure repeats until all conditions are tested. To evaluate model performance, we measure Pearson's correlation coefficient (PCC) between predicted expression levels and average of known expression levels for profiles belonging to the test condition. Not all conditions are "testable" as unknown as any of four types (i.e. strain information, medium, environmental abiotic stresses, and genetic perturbations) representing test condition might not be present in the training set. From the compendium, 262 are "testable" as unknown among 493 conditions. Theses conditions are classified into 6 groups based on presence of genetic perturbations and stresses (**Supplementary Fig. 28**).

Baseline measurement. We defined three baselines for model performance evaluation. The first baseline ("*random baseline*") provides the PCC between the average expression level for each gene calculated from 10 random profiles in the training data, to the expression profiles of the test data. For each comparison we report the "random baseline" calculated from 1000 times of repetitive sampling without replacement of the 10-profile random set. The second baseline ("*mean baseline*") provides the PCC between the average expression level of each gene calculated from all profiles in the training data, to the expression profiles of the test data. The third baseline ("*WT baseline*") provides the PCC between the average expression level for each gene calculated from all WT profiles in the training data,

to the expression profiles of the test data. Again, we define as WT (wild-type) profiles the ones of the MG1655 strain in LB or M9 medium, any carbon source, without any stresses or genetic perturbation.

3.3.4 Proteome module

The expression level for each of the 1,001 proteins is predicted through an ensemble method that integrates information from four sources: the transcriptional regulatory network (TRN), the protein-protein interaction (PPI) network, the co-expressed protein network (CPN) and other pathway information. **Supplementary Fig. 20** depicts the integration among these layers to derive the protein expression level. More specifically:

- **Transcriptional Regulatory Network (TRN).** The transcriptional regulatory network was built based on the `RegulonDB` database as described in **Section 3.1.3**. The protein level of a target gene in a novel condition is predicted by LASSO regression [122] of the expression levels of genes that are connected through a regulatory link to the target gene.
- **Protein-Protein Interaction (PPI) Network.** The PPI network was constructed from the five distinctive sources that were described in **Section 3.1.3**. Similarly, the protein level of a target gene in a novel condition is predicted by LASSO regression of the expression levels of genes whose respective proteins are connected through a Protein-Protein interaction to the target protein.
- **Co-expression protein network (CPN).** The co-expressed network (70,710 interactions of 3,163 proteins was built from the core proteome dataset (**Section 3.1.1**), which represents 20 expression profiles of 1,001 proteins, which are not used for proteome prediction. For two proteins to be considered co-expressed, their pairwise correlation should be larger than 0.7. Any given protein level in a novel condition is predicted by LASSO regression of the protein expression levels of co-expressed proteins with respect to the target protein.
- **Pathway clustering.** We cluster genes that are implicated in the same pathways, as represented in the `KEGG` database. The protein level of a target gene in a novel condition is predicted by LASSO regression of the expression levels of genes that are implicated in the same pathways as the target gene.

Prior to building the prediction models, we apply min-max scaling of the proteome data for preprocessing as in **Section 3.3.3**. The λ L_1-regularization parameter for each linear function is selected based on cross-validation. For evaluating prediction of proteome layer, LOCO cross-validation is used where a profile is left out for testing, the rest is used for training and this procedure repeats until all profiles are tested. PCC between predicted expression levels and known expression levels for all tested profiles is used for a measure of evaluating the prediction performance.

We evaluated each of these methods individually, as well as their integration through an Ensemble method where the protein expression level is the mean of the predicted expression level from each of the four prediction modules. The evaluation was performed in an Ecomics-derived dataset of 18 profiles (5 conditions) with expression levels in both the transcriptional (4,096 transcripts) and proteome layers (1,001 proteins). The integration of four methods has the highest protein coverage and can predict all 1001 proteins, while three of the four individual methods can predict a substantial lower number of proteins (250 for TRN, 547 for KEGG, 1,000 for PPI, 847 for CPN). The prediction performance of the integrated method (0.55$\pm$0.26) outperforms all individual methods (0.41$\pm$0.23 for TRN; 0.47$\pm$0.23 for KEGG; 0.48$\pm$0.26 for PPI; 0.52$\pm$0.24 for CPN). Although we cannot rigorously compare the different PCC among the different methods (they correspond to different sets and numbers of proteins), we can evaluate the protein expression prediction for the top 50 most variable proteins that are common among the five sets. In that case, the Ensemble method that integrates the four different prediction sources

clearly outperforms all other combinations with PCC of 0.77±0.27 while the second best method is CPN-based prediction, with PCC of 0.69±0.27. Additionally, predicting first the target gene expression from the mRNA expression levels of the corresponding genes does not perform well, achieving a PCC of 0.34±0.18 in the general case and PCC of 0.18±0.51 for the 50 most variable proteins (**Supplementary Fig. 20**).

3.3.5 Metabolome module

First, we investigated what information and from which layer (transcriptome, proteome) can lead to a better predictor for the metabolome layer. For this, we used 33 profiles of 126 metabolites, 53 proteins and 75 genes that constitute the core metabolism, in order to predict the concentration of each metabolite. We used i) the expression levels of all 53 proteins and ii) the expression levels of all 75 genes, using constraint LASSO regression in both cases (**Supplementary Fig. 15**). As shown in **Supplementary Fig. 21A**, the prediction performance is better in using (measured) protein expression levels (PCC 0.65±0.21) than by using gene expression levels (PCC 0.47±0.26). For predicting concentrations of metabolites in non-core metabolism, we resort to inferring their levels from mRNA expression levels due to the paucity of profiles with both metabolome (including metabolites in non-core metabolism) and proteome information (only 6 profiles). Variance analysis indicates that the prediction of metabolite concentrations in non-core pathways are robust, with a variance of 0.02±0.01, comparable to that of core metabolism (0.06±0.01), suggesting that such metabolites are highly predictable even without the need of protein expression data (**Supplementary Fig. 21B**). For testing this, we use 115 profiles of 230 metabolites (non-core) and 4,096 genes. For each of metabolites, we train a linear function using LASSO that predicts the concentration of a metabolite from transcriptional expression levels. For metabolites having known enzyme-substrate relations, we predict its concentrations from the mRNA expression levels of the related enzymes. For those with no such information, we fit from all the genes by using LASSO, which allows variable selection. We perform leave-one-out cross-validation from the data set to validate the prediction performance of the approach. The results (**Supplementary Fig. 21C**) show that the prediction performance is PCC 0.87±0.16, although the baseline is also high (PCC 0.77±0.17 for mean baseline and 0.70±0.13 for random baseline) because of the invariance of metabolite concentrations in non-core. Finally, we predict the metabolome layer from two components that i) predicts 126 metabolites from 75 protein expression levels in core metabolism and ii) predicts 230 metabolites from 4096 gene expression levels in non-core metabolism (**Supplementary Fig. 15**).

3.3.6 Fluxome module

Fluxes are predicted using Flux Balance Analysis (FBA), which is a mathematical approach for analyzing the flow of metabolites through a metabolic network [132]. Basically, FBA can be formulated as the following optimization problem:

$$\begin{aligned} &\textbf{maximize} && c^T v \\ &\textbf{subject to} && Sv = 0 \\ &\textbf{and} && l_i \leq v_i \leq u_i \text{ for all } i \end{aligned}$$

where c is a vector of coefficients and v is a reaction vector. S is stoichiometric matrix. We make reaction constraints based on expression levels of related enzymes. Specifically, we change the lower bounds of reactions by the following rule:

$$\begin{cases} l_i = 0 & \text{if } mean(g_i) \leq t \\ l_i = -1000 & \text{otherwise} \end{cases}$$

where g_i is expression levels of enzymes in reaction i (r_i). Threshold t is determined within the range of $\{0.02, 0.04, 0.06, 0.08\}$ that maximizes the prediction performance for growth rate in training data and it was determined to be 0.04. The range was determined to be below 0.1 as the mean expression level of genes was 0.10. For this, GPR (gene-protein-reaction) associations from `BiGG` database [81] are retrieved (iJO1366). For exchange reactions for the metabolites present in LB medium, we followed the reaction bounds from [133]. For anaerobic condition, exchange reaction of oxygen is lower-bounded to zero. Bounds for all exchange reactions are listed in **Supplementary Data 8**. Expression levels of enzymes are interrogated from proteome layer if measured and from transcriptome layer otherwise. If any of enzymes in a reaction are not measured, then lower bound of the reaction is unconstrained. Typically, a FBA solution is not one as multiple genome-wide fluxes might achieves the same objective. From the multiple solutions, we advocate the flux distribution that minimizes total the total absolute flux (MTF) [134] with the same objective value. To have the predicted growth rate from FBA comparable to ones predicted from other layers, we built a linear transformation function between predicted growth rate from FBA and measured growth rate for training conditions.

3.3.7 Model integration and phenome prediction

Predicting growth rate from a single layer We built 3 linear functions using LASSO to predict growth rate from (a) condition features, (b) known expression levels of molecular species, and (c) integration of both. We evaluate the predictive capacity of this method for the three layers of transcriptome, proteome, and metabolome. In our evaluation, we keep only the profiles with exponential phase for which growth rates are present in the compendium. This results to the following datasets:

- Transcriptome: 1,328 profiles of 4,096 genes (111 conditions)
- Proteome: 57 profiles of 410 proteins (33 conditions)
- Metabolome: 577 profiles of 171 metabolites (49 conditions)

The results show that for all layers, the integration of condition information (strain, medium, stress, genetic perturbation) and expression profiles for a single layer predicts more accurately the growth rate than using the condition input and the expression profiles independently (**Supplementary Fig. 27**). The performance of models that solely use condition features is variable across different layers.

Prediction performance of the integrated model. We integrate all layers for a consensus prediction of growth rate by using a weighted sum model, which was found to outperform other candidates in our testing conditions. The final predicted growth rate is the weighted sum of the growth rate predicted in each layer. The weight for each term is the normalized performance on predicting the growth rate for each layer, measured by LOCO cross-validation during the training phase. As such:

$$g_i = \sum_{l}^{L} w_l g_l \tag{5}$$

where g_i is the consensus growth rate from all five layers, L is a set of all five layers, w_l is the weight factor for layer l, g_l is the growth rate predicted from layer l. The normalized training performance for layer l is calculated by PCC between measured growth rate and predicted growth rate from layer l for all

conditions in the training set normalized by sum of PCC for all l. Our results show that the performance of the model integrating all five layers (PCC=0.6) performs better than any other alternative model (**Fig. 22**). The performance increases in cases where wild-type conditions are present in the training set. Additionally, when the model is predicting unknown WT conditions, the PCC increases up to 0.76. We also investigated individual cases to document the effect of each layer to prediction performance. In 53 cases, the distance between predicted and measured growth rate gradually decreases as each layer is supplemented ($R < 0$). As shown in (**Fig. 23**), the largest PCC gains are with the introduction of the transcriptome (increase from PCC=0.46 to 0.64) and fluxome layer (increase from PCC=0.65 to 0.70).

3.4 Experimental Procedures

3.4.1 Targeted experimentation for RNA-Seq

For the compendium enrichment, three replicates of selected *E. coli* mutants **Supplementary Table 2** from the Keio collection [135] were grown in minimal M9 salt medium with 0.4% (w/v) glucose as carbon source. Cells were grown in 3 mL of media till mid stationary phase (approx. 8 hours) and then mixed with 1.5 ml of chilled 5% phenol/ethanol (v/v). After centrifugation, cells were stored at -80C until use, never more than one week. RNA was extracted using the RNeasy kit (Qiagen) and enriched with MICROBExpress (Ambion). RNA fragmentation, cDNA production, A-tailing, linker ligation and PCR enrichment were made using the KAPA Stranded RNA-Seq Library Preparation Kit (Kapa Biosystems). Size selection of the final library was performed with Agencourt AMPure XP (Beckman Coulter). After quality control, libraries were pooled and sequenced using an Illumina HiSeq 2500 sequencer.

3.4.2 Proteome profiling

E. coli MG1655 was grown in M9 with 0.4% glucose with and without 0.6% n-butanol for 12 hours (early stationary). Bacteria were pelleted by centrifugation and the samples were analyzed in the Proteomics Core at Genome Center (UC Davis). The processing steps of protein quantification are described in the main text.

3.4.3 Growth experiments

Strains and media. Growth measurements were performed using different strains of the *E. coli*. *E. coli* strains used were either from our lab collection or were gifted by different research laboratories. *E. coli* strains were preserved in Luria Bertani broth supplemented with 15% glycerol. Specific media were made as per requirement.

Lag phase, maximum growth rate and maximum cell density measurements. For the phenomics layer, the growth curves were experimentally measured (**Supplementary Data 7**) at least in triplicates. 5 μl of the preserved *E. coli* cells were grown overnight in either 1 ml of 0.2% glycerol M9 medium or 1 ml of the required medium (in the case of carbon shift) in an incubator shaker (Innova 44, New Brunswick Scientific) operating at 175 rpm at 37C. Next day, 3 μl of the growing cultures were taken and inoculated into 197 μl of specific media in a 96 well flat bottom plate (Costar). Cells were grown in an incubator shaker (BioTek Synergy HT) operating at the required temperature. Cell densities were measured at every 15 minutes. For anaerobic growth curves, a custom chamber was made, which was saturated with the nitrogen. 96 well plate was placed inside the chamber and 3 μl of the growing cultures were taken and inoculated into 197 μl of required media saturated previously with nitrogen. Plate was sealed using adhesive tape sheets (OmniGenX Microplate Seal Pad) and cell densities were measured at every 15 minutes. An automated script was written in the `MATLAB` to calculate the lag phase, maximum growth rate and maximum cell density. The lag phase was defined as the duration to achieve 5% of the maximum growth rate. Growth rates were determined by calculating the differential between 10 to 90% of cell density using a virtual sliding window with the width of 1 hour and sliding every 15 minutes.

References

[1] Javier Carrera, Raissa Estrela, Jing Luo, Navneet Rai, Athanasios Tsoukalas, and Ilias Tagkopoulos. An integrative, multi-scale, genome-wide model reveals the phenotypic landscape of *Escherichia coli*. *Molecular systems biology*, 10(7), 2014.

[2] Ron Edgar, Michael Domrachev, and Alex E Lash. Gene expression omnibus: Ncbi gene expression and hybridization array data repository. *Nucleic acids research*, 30(1):207–210, 2002.

[3] Alvis Brazma, Helen Parkinson, Ugis Sarkans, Mohammadreza Shojatalab, Jaak Vilo, Niran Abeygunawardena, Ele Holloway, Misha Kapushesky, Patrick Kemmeren, Gonzalo Garcia Lara, et al. Arrayexpress—a public repository for microarray gene expression data at the ebi. *Nucleic acids research*, 31(1):68–71, 2003.

[4] Rasko Leinonen, Hideaki Sugawara, and Martin Shumway. The sequence read archive. *Nucleic acids research*, page gkq1019, 2010.

[5] Philip Jones, Richard G Côté, Lennart Martens, Antony F Quinn, Chris F Taylor, William Derache, Henning Hermjakob, and Rolf Apweiler. Pride: a public repository of protein and peptide identifications for the proteomics community. *Nucleic acids research*, 34(suppl 1):D659–D663, 2006.

[6] Juan A Vizcaíno, Eric W Deutsch, Rui Wang, Attila Csordas, Florian Reisinger, Daniel Rios, Jose A Dianes, Zhi Sun, Terry Farrah, Nuno Bandeira, et al. Proteomexchange provides globally coordinated proteomics data submission and dissemination. *Nature biotechnology*, 32(3):223–226, 2014.

[7] Nobuyoshi Ishii, Kenji Nakahigashi, Tomoya Baba, Martin Robert, Tomoyoshi Soga, Akio Kanai, Takashi Hirasawa, Miki Naba, Kenta Hirai, Aminul Hoque, et al. Multiple high-throughput analyses monitor the response of *E. coli* to perturbations. *Science*, 316(5824):593–597, 2007.

[8] L Arike, K Valgepea, L Peil, R Nahku, K Adamberg, and R Vilu. Comparison and applications of label-free absolute proteome quantification methods on *Escherichia coli*. *Journal of proteomics*, 75(17):5437–5448, 2012.

[9] Peng Lu, Christine Vogel, Rong Wang, Xin Yao, and Edward M Marcotte. Absolute protein expression profiling estimates the relative contributions of transcriptional and translational regulation. *Nature biotechnology*, 25(1):117–124, 2007.

[10] Nathan E Lewis, Kim K Hixson, Tom M Conrad, Joshua A Lerman, Pep Charusanti, Ashoka D Polpitiya, Joshua N Adkins, Gunnar Schramm, Samuel O Purvine, Daniel Lopez-Ferrer, et al. Omic data from evolved *E. coli* are consistent with computed optimal growth from genome-scale models. *Molecular systems biology*, 6(1), 2010.

[11] Kian-Kai Cheng, Baek-Seok Lee, Takeshi Masuda, Takuro Ito, Kazutaka Ikeda, Akiyoshi Hirayama, Lingli Deng, Jiyang Dong, Kazuyuki Shimizu, Tomoyoshi Soga, et al. Global metabolic network reorganization by adaptive mutations allows fast growth of *Escherichia coli* on glycerol. *Nature communications*, 5, 2014.

[12] Sheng Hui, Josh M Silverman, Stephen S Chen, David W Erickson, Markus Basan, Jilong Wang, Terence Hwa, and James R Williamson. Quantitative proteomic analysis reveals a simple strategy of global resource allocation in bacteria. *Molecular systems biology*, 11(2):784, 2015.

[13] Kevin E Eboigbodin and Catherine A Biggs. Characterization of the extracellular polymeric substances produced by *Escherichia coli* using infrared spectroscopic, proteomic, and aggregation studies. *Biomacromolecules*, 9(2):686–695, 2008.

[14] Nelson C Soares, Philipp Spat, Karsten Krug, and Boris Macek. Global dynamics of the *Escherichia coli* proteome and phosphoproteome during growth in minimal medium. *Journal of proteome research*, 12(6):2611–2621, 2013.

[15] Ryosuke L Ohniwa, Yuri Ushijima, Shinji Saito, and Kazuya Morikawa. Proteomic analyses of nucleoid-associated proteins in *Escherichia coli*, *Pseudomonas aeruginosa*, *Bacillus subtilis*, and *Staphylococcus aureus*. *PLoS One*, 6(4):e19172, 2011.

[16] Harry E Pence and Antony Williams. Chemspider: an online chemical information resource. *Journal of Chemical Education*, 87(11):1123–1124, 2010.

[17] David S Wishart, Dan Tzur, Craig Knox, Roman Eisner, An Chi Guo, Nelson Young, Dean Cheng, Kevin Jewell, David Arndt, Summit Sawhney, et al. Hmdb: the human metabolome database. *Nucleic acids research*, 35(suppl 1):D521–D526, 2007.

[18] Yanli Wang, Jewen Xiao, Tugba O Suzek, Jian Zhang, Jiyao Wang, and Stephen H Bryant. Pubchem: a public information system for analyzing bioactivities of small molecules. *Nucleic acids research*, 37(suppl 2):W623–W633, 2009.

[19] Bryson D Bennett, Elizabeth H Kimball, Melissa Gao, Robin Osterhout, Stephen J Van Dien, and Joshua D Rabinowitz. Absolute metabolite concentrations and implied enzyme active site occupancy in escherichia coli. *Nature chemical biology*, 5(8):593–599, 2009.

[20] Dinesh Kumar Barupal, Sang Jun Lee, Edward D Karoly, and Sankar Adhya. Inactivation of metabolic genes causes short-and long-range dys-regulation in *Escherichia coli* metabolic network. *PloS one*, 8(12):e78360, 2013.

[21] Yi-Fan Xu, Daniel Amador-Noguez, Marshall Louis Reaves, Xiao-Jiang Feng, and Joshua D Rabinowitz. Ultrasensitive regulation of anapleurosis via allosteric activation of pep carboxylase. *Nature chemical biology*, 8(6):562–568, 2012.

[22] Szymon Jozefczuk, Sebastian Klie, Gareth Catchpole, Jedrzej Szymanski, Alvaro Cuadros-Inostroza, Dirk Steinhauser, Joachim Selbig, and Lothar Willmitzer. Metabolomic and transcriptomic stress response of *Escherichia coli*. *Molecular systems biology*, 6(1), 2010.

[23] Jochen Schaub, Klaus Mauch, and Matthias Reuss. Metabolic flux analysis in *Escherichia coli* by integrating isotopic dynamic and isotopic stationary 13c labeling data. *Biotechnology and Bioengineering*, 99(5):1170–1185, 2008.

[24] Hilal Taymaz-Nikerel, Marjan De Mey, Cor Ras, Angela ten Pierick, Reza M Seifar, Jan C Van Dam, Joseph J Heijnen, and Walter M van Gulik. Development and application of a differential method for reliable metabolome analysis in *Escherichia coli*. *Analytical biochemistry*, 386(1):9–19, 2009.

[25] Christoph Wittmann, Jan Weber, Eriola Betiku, Jens Krömer, Daniela Böhm, and Ursula Rinas. Response of fluxome and metabolome to temperature-induced recombinant protein synthesis in *Escherichia coli*. *Journal of biotechnology*, 132(4):375–384, 2007.

[26] Anders K Holm, Lars M Blank, Marco Oldiges, Andreas Schmid, Christian Solem, Peter R Jensen, and Goutham N Vemuri. Metabolic and transcriptional response to cofactor perturbations in *Escherichia coli*. *Journal of Biological Chemistry*, 285(23):17498–17506, 2010.

[27] Yoshihiro Toya, Kenji Nakahigashi, Masaru Tomita, and Kazuyuki Shimizu. Metabolic regulation analysis of wild-type and *arcA* mutant *Escherichia coli* under nitrate conditions using different levels of omics data. *Molecular BioSystems*, 8(10):2593–2604, 2012.

[28] Scott B Crown, Christopher P Long, and Maciek R Antoniewicz. Integrated 13 c-metabolic flux analysis of 14 parallel labeling experiments in *Escherichia coli*. *Metabolic engineering*, 2015.

[29] Lisa M Maurer, Elizabeth Yohannes, Sandra S Bondurant, Michael Radmacher, and Joan L Slonczewski. ph regulates genes for flagellar motility, catabolism, and oxidative stress in *Escherichia coli* k-12. *Journal of bacteriology*, 187(1):304–319, 2005.

[30] Janet Flatley, Jason Barrett, Steven T Pullan, Martin N Hughes, Jeffrey Green, and Robert K Poole. Transcriptional responses of *Escherichia coli* to s-nitrosoglutathione under defined chemostat conditions reveal major changes in methionine biosynthesis. *Journal of Biological Chemistry*, 280(11):10065–10072, 2005.

[31] Fu'ad T Haddadin and Sarah W Harcum. Transcriptome profiles for high-cell-density recombinant and wild-type *Escherichia coli*. *Biotechnology and bioengineering*, 90(2):127–153, 2005.

[32] Christopher J Kershaw, Nigel L Brown, Chrystala Constantinidou, Mala D Patel, and Jon L Hobman. The expression profile of *Escherichia coli* k-12 in response to minimal, optimal and excess copper concentrations. *Microbiology*, 151(4):1187–1198, 2005.

[33] Katy C Kao, Linh M Tran, and James C Liao. A global regulatory role of gluconeogenic genes in *Escherichia coli* revealed by transcriptome network analysis. *Journal of Biological Chemistry*, 280(43):36079–36087, 2005.

[34] Christopher A Elkins and Lisa B Mullis. Mammalian steroid hormones are substrates for the major rnd-and mfs-type tripartite multidrug efflux pumps of *Escherichia coli*. *Journal of bacteriology*, 188(3):1191–1195, 2006.

[35] Matthew F Traxler, Dong-Eun Chang, and Tyrrell Conway. Guanosine 3', 5'-bispyrophosphate coordinates global gene expression during glucose-lactose diauxie in *Escherichia coli*. *Proceedings of the National Academy of Sciences of the United States of America*, 103(7):2374–2379, 2006.

[36] Michael J Allen, Graham F White, and Andrew P Morby. The response of *Escherichia coli* to exposure to the biocide polyhexamethylene biguanide. *Microbiology*, 152(4):989–1000, 2006.

[37] C Bianco, E Imperlini, R Calogero, B Senatore, P Pucci, and R Defez. Indole-3-acetic acid regulates the central metabolic pathways in *Escherichia coli*. *Microbiology*, 152(8):2421–2431, 2006.

[38] Everett T Hayes, Jessica C Wilks, Piero Sanfilippo, Elizabeth Yohannes, Daniel P Tate, Brian D Jones, Michael D Radmacher, Sandra S BonDurant, and Joan L Slonczewski. Oxygen limitation modulates ph regulation of catabolism and hydrogenases, multidrug transporters, and envelope composition in *Escherichia coli* k-12. *BMC microbiology*, 6(1):89, 2006.

[39] Jeremiah J Faith, Boris Hayete, Joshua T Thaden, Ilaria Mogno, Jamey Wierzbowski, Guillaume Cottarel, Simon Kasif, James J Collins, and Timothy S Gardner. Large-scale mapping and validation of *Escherichia coli* transcriptional regulation from a compendium of expression profiles. *PLoS biology*, 5(1):e8, 2007.

[40] Toshinari Maeda, Gönül Vardar, William T Self, and Thomas K Wood. Inhibition of hydrogen uptake in *Escherichia coli* by expressing the hydrogenase from the cyanobacterium *Synechocystis* sp. pcc 6803. *BMC biotechnology*, 7(1):25, 2007.

[41] Jeffrey L Blanchard, Wei-Yun Wholey, Erin M Conlon, and Pablo J Pomposiello. Rapid changes in gene expression dynamics in response to superoxide reveal soxrs-dependent and independent transcriptional networks. *PLoS One*, 2(11):e1186, 2007.

[42] Mary E Laubacher and Sarah E Ades. The rcs phosphorelay is a cell envelope stress response activated by peptidoglycan stress and contributes to intrinsic antibiotic resistance. *Journal of bacteriology*, 190(6):2065–2074, 2008.

[43] Thusitha S Gunasekera, Laszlo N Csonka, and Oleg Paliy. Genome-wide transcriptional responses of *Escherichia coli* k-12 to continuous osmotic and heat stresses. *Journal of bacteriology*, 190(10):3712–3720, 2008.

[44] Matthew F Traxler, Sean M Summers, Huyen-Tran Nguyen, Vineetha M Zacharia, G Aaron Hightower, Joel T Smith, and Tyrrell Conway. The global, ppgpp-mediated stringent response to amino acid starvation in *Escherichia coli*. *Molecular microbiology*, 68(5):1128–1148, 2008.

[45] Christopher J Cardinale, Robert S Washburn, Vasisht R Tadigotla, Lewis M Brown, Max E Gottesman, and Evgeny Nudler. Termination factor rho and its cofactors nusa and nusg silence foreign dna in *E. coli*. *Science*, 320(5878):935–938, 2008.

[46] Jintae Lee, Xue-Song Zhang, Manjunath Hegde, William E Bentley, Arul Jayaraman, and Thomas K Wood. Indole cell signaling occurs primarily at low temperatures in *Escherichia coli*. *The ISME journal*, 2(10):1007–1023, 2008.

[47] Rodolfo García-Contreras, Xue-Song Zhang, Younghoon Kim, and Thomas K Wood. Protein translation and cell death: the role of rare trnas in biofilm formation and in activating dormant phage killer genes. *PLoS One*, 3(6):e2394–e2394, 2008.

[48] T Haddadin Fu'ad, Harry Kurtz, and Sarah W Harcum. Serine hydroxamate and the transcriptome of high cell density recombinant *Escherichia coli* mg1655. *Applied biochemistry and biotechnology*, 157(2):124–139, 2009.

[49] Lana Shabala, John Bowman, Janelle Brown, Tom Ross, Tom McMeekin, and Sergey Shabala. Ion transport and osmotic adjustment in *Escherichia coli* in response to ionic and non-ionic osmotica. *Environmental microbiology*, 11(1):137–148, 2009.

[50] K Lemuth, T Hardiman, S Winter, D Pfeiffer, MA Keller, S Lange, M Reuss, RD Schmid, and M Siemann-Herzberg. Global transcription and metabolic flux analysis of *Escherichia coli* in glucose-limited fed-batch cultivations. *Applied and environmental microbiology*, 74(22):7002–7015, 2008.

[51] Jaakko Soini, Christina Falschlehner, Christina Liedert, Jörg Bernhardt, Jussi Vuoristo, and Peter Neubauer. Norvaline is accumulated after a down-shift of oxygen in *Escherichia coli* w3110. *Microb Cell Fact*, 7:30, 2008.

[52] Kelly S Davidge, Guido Sanguinetti, Chu Hoi Yee, Alan G Cox, Cameron W McLeod, Claire E Monk, Brian E Mann, Roberto Motterlini, and Robert K Poole. Carbon monoxide-releasing antibacterial molecules target respiration and global transcriptional regulators. *Journal of Biological Chemistry*, 284(7):4516–4524, 2009.

[53] Lígia S Nobre, Fatima Al-Shahrour, Joaquin Dopazo, and Lígia M Saraiva. Exploring the antimicrobial action of a carbon monoxide-releasing compound through whole-genome transcription profiling of *Escherichia coli*. *Microbiology*, 155(3):813–824, 2009.

[54] GO Thomassen, Alexander D Rowe, Karin Lagesen, Jessica M Lindvall, and Torbjørn Rognes. Custom design and analysis of high-density oligonucleotide bacterial tiling microarrays. *PloS one*, 4(6):e5943, 2009.

[55] William A Glover, Yanqin Yang, and Ying Zhang. Insights into the molecular basis of l-form formation and survival in *Escherichia coli*. *PLoS One*, 4(10):e7316, 2009.

[56] Kun Zhu, Yong-Mei Zhang, and Charles O Rock. Transcriptional regulation of membrane lipid homeostasis in *Escherichia coli*. *Journal of Biological Chemistry*, 284(50):34880–34888, 2009.

[57] Byung-Kwan Cho, Karsten Zengler, Yu Qiu, Young Seoub Park, Eric M Knight, Christian L Barrett, Yuan Gao, and Bernhard Ø Palsson. The transcription unit architecture of the *Escherichia coli* genome. *Nature biotechnology*, 27(11):1043–1049, 2009.

[58] J Lee, SR Hiibel, KF Reardon, and TK Wood. Identification of stress-related proteins in *Escherichia coli* using the pollutant cis-dichloroethylene. *Journal of applied microbiology*, 108(6):2088–2102, 2010.

[59] Dipen P Sangurdekar, Bree L Hamann, Dmitri Smirnov, Friedrich Srienc, Philip C Hanawalt, and Arkady B Khodursky. Thymineless death is associated with loss of essential genetic information from the replication origin. *Molecular microbiology*, 75(6):1455–1467, 2010.

[60] Xiaoxue Wang, Younghoon Kim, Qun Ma, Seok Hoon Hong, Karina Pokusaeva, Joseph M Sturino, and Thomas K Wood. Cryptic prophages help bacteria cope with adverse environments. *Nature communications*, 1:147, 2010.

[61] Jeremy J Minty, Ann A Lesnefsky, Fengming Lin, Yu Chen, Ted A Zaroff, Artur B Veloso, Bin Xie, Catie A McConnell, Rebecca J Ward, Donald R Schwartz, et al. Evolution combined with genomic study elucidates genetic bases of isobutanol tolerance in *Escherichia coli*. *Microb Cell Fact*, 10(18):10–1186, 2011.

[62] Anna Stincone, Nazish Daudi, Ayesha S Rahman, Philipp Antczak, Ian Henderson, Jeffrey Cole, Matthew D Johnson, Peter Lund, and Francesco Falciani. A systems biology approach sheds new light on *Escherichia coli* acid resistance. *Nucleic acids research*, 39(17):7512–7528, 2011.

[63] Rebecca M Lennen, Max A Kruziki, Kritika Kumar, Robert A Zinkel, Kristin E Burnum, Mary S Lipton, Spencer W Hoover, Don R Ranatunga, Tyler M Wittkopp, Wesley D Marner, et al. Membrane stresses induced by overproduction of free fatty acids in *Escherichia coli*. *Applied and environmental microbiology*, 77(22):8114–8128, 2011.

[64] Dongping Wang, Bernarda Calla, Sornkanok Vimolmangkang, Xia Wu, Schuyler S Korban, Steven C Huber, Steven J Clough, and Youfu Zhao. The orphan gene *ybjN* conveys pleiotropic effects on multicellular behavior and survival of *Escherichia coli*. *PLoS One*, 6(9):e25293, 2011.

[65] Dipen P Sangurdekar, Zhigang Zhang, and Arkady B Khodursky. The association of dna damage response and nucleotide level modulation with the antibacterial mechanism of the anti-folate drug trimethoprim. *BMC genomics*, 12(1):583, 2011.

[66] Mirjana Macvanin, Rotem Edgar, Feng Cui, Andrei Trostel, Victor Zhurkin, and Sankar Adhya. Noncoding rnas binding to the nucleoid protein hu in *Escherichia coli*. *Journal of bacteriology*, 194(22):6046–6055, 2012.

[67] Wei Lu, Liang Li, Ming Chen, Zhengfu Zhou, Wei Zhang, Shuzhen Ping, Yongliang Yan, Jin Wang, and Min Lin. Genome-wide transcriptional responses of *Escherichia coli* to glyphosate, a potent inhibitor of the shikimate pathway enzyme 5-enolpyruvylshikimate-3-phosphate synthase. *Molecular BioSystems*, 9(3):522–530, 2013.

[68] Simona Kamenšek and Darja Žgur-Bertok. Global transcriptional responses to the bacteriocin colicin m in *Escherichia coli*. *BMC microbiology*, 13(1):42, 2013.

[69] Kotakonda Arunasri, Mohammed Adil, Katari Venu Charan, Chatterjee Suvro, Seerapu Himabindu Reddy, and Sisinthy Shivaji. Effect of simulated microgravity on *E. coli* k12 mg1655 growth and gene expression. *PloS one*, 8(3):e57860, 2013.

[70] Samantha McLean, Ronald Begg, Helen E Jesse, Brian E Mann, Guido Sanguinetti, and Robert K Poole. Analysis of the bacterial response to ru (co) 3cl (glycinate)(corm-3) and the inactivated compound identifies the role played by the ruthenium compound and reveals sulfur-containing species as a major target of corm-3 action. *Antioxidants & redox signaling*, 19(17):1999–2012, 2013.

[71] Nadine Händel, J Merijn Schuurmans, Stanley Brul, and Benno H ter Kuile. Compensation of the metabolic costs of antibiotic resistance by physiological adaptation in *Escherichia coli*. *Antimicrobial agents and chemotherapy*, pages AAC–02096, 2013.

[72] Ryan McClure, Divya Balasubramanian, Yan Sun, Maksym Bobrovskyy, Paul Sumby, Caroline A Genco, Carin K Vanderpool, and Brian Tjaden. Computational analysis of bacterial rna-seq data. *Nucleic acids research*, 41(14):e140–e140, 2013.

[73] Wei Liu, Shi Lei Dong, Fei Xu, Xue Qin Wang, T Ryan Withers, D Yu Hongwei, and Xin Wang. Effect of intracellular expression of antimicrobial peptide ll-37 on growth of *Escherichia coli* strain top10 under aerobic and anaerobic conditions. *Antimicrobial agents and chemotherapy*, 57(10):4707–4716, 2013.

[74] Robert H Dahl, Fuzhong Zhang, Jorge Alonso-Gutierrez, Edward Baidoo, Tanveer S Batth, Alyssa M Redding-Johanson, Christopher J Petzold, Aindrila Mukhopadhyay, Taek Soon Lee, Paul D Adams, et al. Engineering dynamic pathway regulation using stress-response promoters. *Nature biotechnology*, 31(11):1039–1046, 2013.

[75] Roberto C Molina-Quiroz, David E Loyola, Claudia M Muñoz-Villagrán, Raquel Quatrini, Claudio C Vásquez, and José M Pérez-Donoso. Dna, cell wall and general oxidative damage underlie the tellurite/cefotaxime synergistic effect in *Escherichia coli*. 2013.

[76] Liam A Royce, Erin Boggess, Yao Fu, Ping Liu, Jacqueline V Shanks, Julie Dickerson, and Laura R Jarboe. Transcriptomic analysis of carboxylic acid challenge in *Escherichia coli*: beyond membrane damage. *PloS one*, 9(2):e89580, 2014.

[77] Lisa Carraro, Luca Fasolato, Filomena Montemurro, Maria Elena Martino, Stefania Balzan, Maurizio Servili, Enrico Novelli, and Barbara Cardazzo. Polyphenols from olive mill waste affect biofilm formation and motility in *Escherichia coli* k-12. *Microbial biotechnology*, 7(3):265–275, 2014.

[78] Mary B Berlyn and Stanley Letovsky. Genome-related datasets within the *E. coli* genetic stock center database. *Nucleic acids research*, 20(23):6143–6151, 1992.

[79] Brenley K McIntosh, Daniel P Renfro, Gwendowlyn S Knapp, Chanchala R Lairikyengbam, Nathan M Liles, Lili Niu, Amanda M Supak, Anand Venkatraman, Adrienne E Zweifel, Deborah A Siegele, et al. Ecoliwiki: a wiki-based community resource for *Escherichia coli*. *Nucleic acids research*, page gkr880, 2011.

[80] Ingrid M Keseler, Julio Collado-Vides, Socorro Gama-Castro, John Ingraham, Suzanne Paley, Ian T Paulsen, Martín Peralta-Gil, and Peter D Karp. Ecocyc: a comprehensive database resource for *Escherichia coli*. *Nucleic acids research*, 33(suppl 1):D334–D337, 2005.

[81] Jan Schellenberger, Junyoung O Park, Tom M Conrad, and Bernhard Ø Palsson. Bigg: a biochemical genetic and genomic knowledgebase of large scale metabolic reconstructions. *BMC bioinformatics*, 11(1):213, 2010.

[82] Chong Su, Jose M Peregrin-Alvarez, Gareth Butland, Sadhna Phanse, Vincent Fong, Andrew Emili, and John Parkinson. Bacteriome. org—an integrated protein interaction database for *E. coli*. *Nucleic acids research*, 36(suppl 1):D632–D636, 2008.

[83] Damian Szklarczyk, Andrea Franceschini, Michael Kuhn, Milan Simonovic, Alexander Roth, Pablo Minguez, Tobias Doerks, Manuel Stark, Jean Muller, Peer Bork, et al. The string database in 2011: functional interaction networks of proteins, globally integrated and scored. *Nucleic acids research*, 39(suppl 1):D561–D568, 2011.

[84] Ulrich Stelzl, Uwe Worm, Maciej Lalowski, Christian Haenig, Felix H Brembeck, Heike Goehler, Martin Stroedicke, Martina Zenkner, Anke Schoenherr, Susanne Koeppen, et al. A human protein-protein interaction network: a resource for annotating the proteome. *Cell*, 122(6):957–968, 2005.

[85] Gareth Butland, José Manuel Peregrín-Alvarez, Joyce Li, Wehong Yang, Xiaochun Yang, Veronica Canadien, Andrei Starostine, Dawn Richards, Bryan Beattie, Nevan Krogan, et al. Interaction network containing conserved and essential protein complexes in *Escherichia coli*. *Nature*, 433(7025):531–537, 2005.

[86] Pingzhao Hu, Sarath Chandra Janga, Mohan Babu, J Javier Díaz-Mejía, Gareth Butland, Wenhong Yang, Oxana Pogoutse, Xinghua Guo, Sadhna Phanse, Peter Wong, et al. Global functional atlas of *Escherichia coli* encompassing previously uncharacterized proteins. *PLoS biology*, 7(4):e1000096, 2009.

[87] Seesandra V Rajagopala, Patricia Sikorski, Ashwani Kumar, Roberto Mosca, James Vlasblom, Roland Arnold, Jonathan Franca-Koh, Suman B Pakala, Sadhna Phanse, Arnaud Ceol, et al. The binary protein-protein interaction landscape of *Escherichia coli*. *Nature biotechnology*, 32(3):285–290, 2014.

[88] Heladia Salgado, Martin Peralta-Gil, Socorro Gama-Castro, Alberto Santos-Zavaleta, Luis Muñiz-Rascado, Jair S García-Sotelo, Verena Weiss, Hilda Solano-Lira, Irma Martínez-Flores, Alejandra Medina-Rivera, et al. Regulondb v8. 0: omics data sets, evolutionary conservation, regulatory phrases, cross-validated gold standards and more. *Nucleic acids research*, 41(D1):D203–D213, 2013.

[89] Byung-Kwan Cho, Donghyuk Kim, Eric M Knight, Karsten Zengler, and Bernhard O Palsson. Genome-scale reconstruction of the sigma factor network in *Escherichia coli*: topology and functional states. *BMC biology*, 12(1):4, 2014.

[90] Laurel Cooper. Common reference ontologies for plant biology (crop): A platform for integrative plant genomics. In *Plant and Animal Genome XXII Conference*. Plant and Animal Genome, 2014.

[91] Sang Woo Seo, Donghyuk Kim, Haythem Latif, Edward J O'Brien, Richard Szubin, and Bernhard O Palsson. Deciphering fur transcriptional regulatory network highlights its complex role beyond iron metabolism in *Escherichia coli*. *Nature communications*, 5, 2014.

[92] Lin He and Gregory J Hannon. Micrornas: small rnas with a big role in gene regulation. *Nature Reviews Genetics*, 5(7):522–531, 2004.

[93] Clayton C Caswell, Amanda G Oglesby-Sherrouse, and Erin R Murphy. Sibling rivalry: related bacterial small rnas and their redundant and non-redundant roles. *Frontiers in cellular and infection microbiology*, 4, 2014.

[94] Danny Ionescu, Björn Voss, Aharon Oren, Wolfgang R Hess, and Alicia M Muro-Pastor. Heterocyst-specific transcription of nsir1, a non-coding rna encoded in a tandem array of direct repeats in cyanobacteria. *Journal of molecular biology*, 398(2):177–188, 2010.

[95] Lauren S Waters and Gisela Storz. Regulatory rnas in bacteria. *Cell*, 136(4):615–628, 2009.

[96] Marc Güell, Eva Yus, Maria Lluch-Senar, and Luis Serrano. Bacterial transcriptomics: what is beyond the rna horiz-ome? *Nature Reviews Microbiology*, 9(9):658–669, 2011.

[97] Yishai Shimoni, Gilgi Friedlander, Guy Hetzroni, Gali Niv, Shoshy Altuvia, Ofer Biham, and Hanah Margalit. Regulation of gene expression by small non-coding rnas: a quantitative view. *Molecular Systems Biology*, 3(1), 2007.

[98] Sabine Brantl. Small regulatory rnas (srnas): key players in prokaryotic metabolism, stress response, and virulence. In *Regulatory RNAs*, pages 73–109. Springer, 2012.

[99] Pankaj Mehta, Sidhartha Goyal, and Ned S Wingreen. A quantitative comparison of srna-based and protein-based gene regulation. *Molecular systems biology*, 4(1), 2008.

[100] Chase L Beisel and Gisela Storz. Base pairing small rnas and their roles in global regulatory networks. *FEMS microbiology reviews*, 34(5):866–882, 2010.

[101] Sahadevan Raman, Taeksun Song, Xiaoling Puyang, Stoyan Bardarov, William R Jacobs, and Robert N Husson. The alternative sigma factor sigh regulates major components of oxidative and heat stress responses in *Mycobacterium tuberculosis*. *Journal of bacteriology*, 183(20):6119–6125, 2001.

[102] Kathryn J Boor. Bacterial stress responses: what doesn't kill them can make them stronger. *PLoS biology*, 4(1):e23, 2006.

[103] Clint Coleman, Chasity Baker, and Cheryl A Nickerson. The role of sigma factors in regulating bacterial stress responses and pathogenesis. In *Molecular Paradigms of Infectious Disease*, pages 438–501. Springer, 2006.

[104] S Wuchty and Peter Uetz. Protein-protein interaction networks of *E. coli* and *S. cerevisiae* are similar. *Scientific reports*, 4, 2014.

[105] Michael B Eisen, Paul T Spellman, Patrick O Brown, and David Botstein. Cluster analysis and display of genome-wide expression patterns. *Proceedings of the National Academy of Sciences*, 95(25):14863–14868, 1998.

[106] Jiawei Han, Micheline Kamber, and Jian Pei. *Data mining, southeast asia edition: Concepts and techniques*. Morgan kaufmann, 2006.

[107] Arthur B Pardee, François Jacob, and Jacques Monod. The genetic control and cytoplasmic expression of "inducibility" in the synthesis of β-galactosidase by *E. coli*. *Journal of Molecular Biology*, 1(2):165–178, 1959.

[108] G Bertani. Studies on lysogenesis i.: The mode of phage liberation by lysogenic *Escherichia coli*. *Journal of bacteriology*, 62(3):293, 1951.

[109] Frederick C Neidhardt, Philip L Bloch, and David F Smith. Culture medium for enterobacteria. *Journal of bacteriology*, 119(3):736–747, 1974.

[110] Michael I Love, Wolfgang Huber, and Simon Anders. Moderated estimation of fold change and dispersion for rna-seq data with deseq2. *Genome Biol*, 15(12):550, 2014.

[111] Sang Jun Lee, Andrei Trostel, Phuoc Le, Rajendran Harinarayanan, Peter C FitzGerald, and Sankar Adhya. Cellular stress created by intermediary metabolite imbalances. *Proceedings of the National Academy of Sciences*, 106(46):19515–19520, 2009.

[112] Hilda Rodrıguez and Reynaldo Fraga. Phosphate solubilizing bacteria and their role in plant growth promotion. *Biotechnology advances*, 17(4):319–339, 1999.

[113] Claudia Sissi and Manlio Palumbo. Effects of magnesium and related divalent metal ions in topoisomerase structure and function. *Nucleic acids research*, 37(3):702–711, 2009.

[114] Mark J Mandel and Thomas J Silhavy. Starvation for different nutrients in escherichia coli results in differential modulation of rpos levels and stability. *Journal of bacteriology*, 187(2):434–442, 2005.

[115] Eric Guisbert, Christophe Herman, Chi Zen Lu, and Carol A Gross. A chaperone network controls the heat shock response in e. coli. *Genes & development*, 18(22):2812–2821, 2004.

[116] John W Foster. Escherichia coli acid resistance: tales of an amateur acidophile. *Nature Reviews Microbiology*, 2(11):898–907, 2004.

[117] Edward W Hickey and Irvin N Hirshfield. Low-ph-induced effects on patterns of protein synthesis and on internal ph in escherichia coli and salmonella typhimurium. *Applied and environmental microbiology*, 56(4):1038–1045, 1990.

[118] Alondra Díaz-Acosta, María L Sandoval, Luis Delgado-Olivares, and Jorge Membrillo-Hernández. Effect of anaerobic and stationary phase growth conditions on the heat shock and oxidative stress responses in escherichia coli k-12. *Archives of microbiology*, 185(6):429–438, 2006.

[119] Jeanne Bonomo and Ryan T Gill. Amino acid content of recombinant proteins influences the metabolic burden response. *Biotechnology and bioengineering*, 90(1):116–126, 2005.

[120] Nicolaos Karayiannis and Anastasios N Venetsanopoulos. *Artificial neural networks: learning algorithms, performance evaluation, and applications*, volume 209. Springer Science & Business Media, 2013.

[121] Johan AK Suykens, Joos PL Vandewalle, and Bart L de Moor. *Artificial neural networks for modelling and control of non-linear systems*. Springer Science & Business Media, 2012.

[122] Robert Tibshirani. Regression shrinkage and selection via the lasso. *Journal of the Royal Statistical Society. Series B (Methodological)*, pages 267–288, 1996.

[123] Jerome Friedman, Trevor Hastie, and Rob Tibshirani. glmnet: Lasso and elastic-net regularized generalized linear models. *R package version*, 1, 2009.

[124] Ken-ichi Funahashi and Yuichi Nakamura. Approximation of dynamical systems by continuous time recurrent neural networks. *Neural networks*, 6(6):801–806, 1993.

[125] James Martens and Ilya Sutskever. Learning recurrent neural networks with hessian-free optimization. In *Proceedings of the 28th International Conference on Machine Learning (ICML-11)*, pages 1033–1040, 2011.

[126] Ilya Sutskever, James Martens, and Geoffrey E Hinton. Generating text with recurrent neural networks. In *Proceedings of the 28th International Conference on Machine Learning (ICML-11)*, pages 1017–1024, 2011.

[127] Rui Xu, Donald Wunsch II, and Ronald Frank. Inference of genetic regulatory networks with recurrent neural network models using particle swarm optimization. *IEEE/ACM Transactions on Computational Biology and Bioinformatics (TCBB)*, 4(4):681–692, 2007.

[128] MW Gardner and SR Dorling. Artificial neural networks (the multilayer perceptron)—a review of applications in the atmospheric sciences. *Atmospheric environment*, 32(14):2627–2636, 1998.

[129] Martin T Hagan, Howard B Demuth, Mark H Beale, et al. *Neural network design*. Pws Pub. Boston, 1996.

[130] Shun-ichi Amari. Backpropagation and stochastic gradient descent method. *Neurocomputing*, 5(4):185–196, 1993.

[131] Federico Girosi, Michael Jones, and Tomaso Poggio. Regularization theory and neural networks architectures. *Neural computation*, 7(2):219–269, 1995.

[132] Jeffrey D Orth, Ines Thiele, and Bernhard Ø Palsson. What is flux balance analysis? *Nature biotechnology*, 28(3):245–248, 2010.

[133] R Mahadevan and CH Schilling. The effects of alternate optimal solutions in constraint-based genome-scale metabolic models. *Metabolic engineering*, 5(4):264–276, 2003.

[134] Robert Schuetz, Nicola Zamboni, Mattia Zampieri, Matthias Heinemann, and Uwe Sauer. Multi-dimensional optimality of microbial metabolism. *Science*, 336(6081):601–604, 2012.

[135] Tomoya Baba, Takeshi Ara, Miki Hasegawa, Yuki Takai, Yoshiko Okumura, Miki Baba, Kirill A Datsenko, Masaru Tomita, Barry L Wanner, and Hirotada Mori. Construction of *Escherichia coli* k-12 in-frame, single-gene knockout mutants: the keio collection. *Molecular systems biology*, 2(1), 2006.
